# Histopathology and spatial transcriptomics jointly map myofiber-specific pathological programs in mTORC1-driven myopathy

**DOI:** 10.1172/jci.insight.203167

**Published:** 2026-08-20

**Authors:** Jer-En Hsu, Qingyang Zhao, Weiqiu Cheng, Hyun Min Kang, Susan V. Brooks, Myungjin Kim, Jun Hee Lee

**Affiliations:** 1 Department of Molecular & Integrative Physiology and; 2 Department of Biostatistics, University of Michigan, Ann Arbor, Michigan, USA.

**Keywords:** Cell biology, Muscle biology, Autophagy, Skeletal muscle, Transcriptomics

## Abstract

Skeletal muscle is composed of heterogeneous myofiber types and non-myocyte populations. Myopathies occur in many diseases, but the mechanisms driving these pathologies remain largely unknown, partly because conventional approaches cannot link histopathological features to molecular states at single-fiber resolution. To address this challenge, we brought histopathology and spatial transcriptomics together by applying high-resolution Seq-Scope technology to a mouse model of mTORC1 hyperactivation. Cross-sections from the extensor digitorum longus (EDL) and soleus (SOL), two muscles with distinct fiber-type compositions, were profiled to determine how transcriptome changes are linked to histopathological outcomes. mTORC1 hyperactivation elicited distinct, fiber type–dependent pathological programs. Type I and IIa fibers were largely resistant to mTORC1-induced pathology, exhibiting relatively limited morphological alterations. In contrast, type IIx fibers diverged into opposing fates: in SOL, they underwent abnormal enlargement associated with sustained growth signaling, cytoskeletal remodeling, and impaired proteostasis; in EDL, they developed basophilia associated with increased RNA content and lipid-, oxidative-, and nucleotide metabolism–related signatures. Within EDL, type IIb fibers displayed heterogeneity with discrete transcriptional states. Non-myocytic populations, including macrophages and fibroblasts, accumulated preferentially in SOL, forming a fibrotic microenvironment associated with inflammation, remodeling, and hypertrophy. These findings provide a link between histopathological phenotypes and molecular states at single-fiber resolution.

## Abstract 2

A high-resolution gene-mapping method links microscopic muscle damage to molecular changes, revealing why different muscle fiber types respond differently to overactive growth signaling.

## Graphical abstract

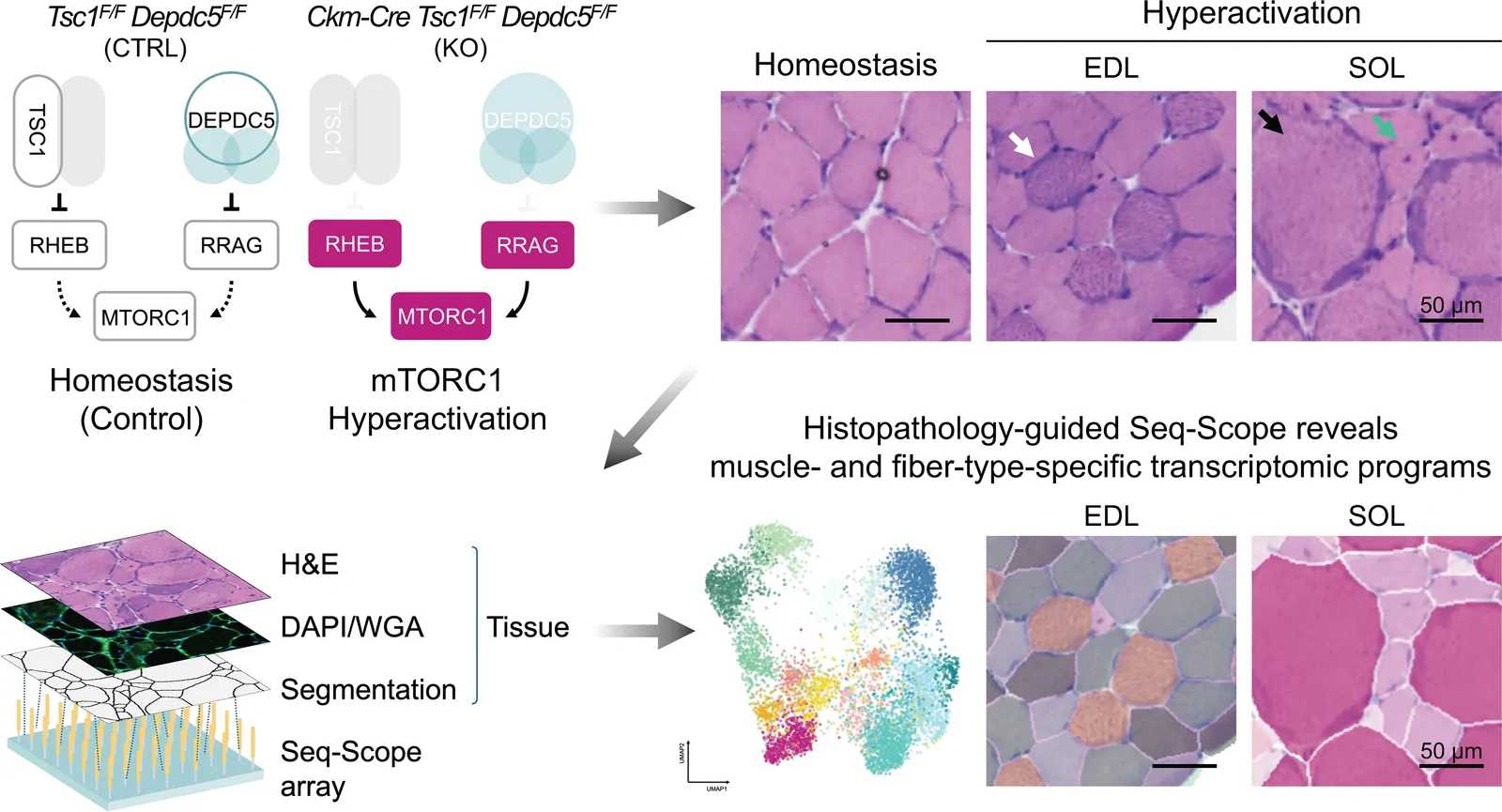

## Introduction

Skeletal muscle is a multifunctional organ that drives movement while being critical for maintaining metabolic homeostasis. This balanced control of essential functions is critical for maintaining health and relies on the heterogeneity of myofibers with respect to contraction and energy metabolism. Differences between myofibers underlie the classic classification into slow and fast and oxidative and glycolytic fiber types, a framework that has long explained variation in endurance, contractile speed, and fatigue resistance (1). At the molecular level, this classification is largely determined by the expression of distinct myosin heavy chain isoforms, which define the major fiber types: type I (MF1; slow oxidative), type IIa (MF2A; fast oxidative), type IIx (MF2X; intermediate fast glycolytic), and type IIb (MF2B; fast glycolytic). The functional specialization of each fiber type allows skeletal muscle as a whole to support diverse physiological demands (2).

mTORC1 is a central regulator of cell growth and anabolism that senses inputs from nutrient, growth factor, and energy signals (3). Its kinase activity is activated at the lysosomal surface through the small GTPase RHEB, which is inhibited by the TSC1/TSC2 complex during growth factor deprivation or energy depletion. In parallel, mTORC1 recruitment to lysosomes is controlled by amino acid availability. Under amino acid insufficiency, the GATOR1 complex (DEPDC5, NPRL2, and NPRL3) maintains the Rag GTPases (RRAGA/B and RRAGC/D) in their inactive GDP-bound state, thereby preventing mTORC1 from translocating to the lysosomal surface and becoming activated (4, 5). Disruption of either TSC1 or DEPDC5 elevates mTORC1 activity, but full constitutive activation requires disruption of both pathways due to the presence of negative feedback mechanisms (6–8). Once active, mTORC1 phosphorylates downstream targets such as S6K1 and 4E-BP1 to promote mRNA translation, while simultaneously suppressing autophagy initiation through inhibitory phosphorylation of ULK1 (9–11). By linking environmental inputs to biosynthetic outputs, mTORC1 ensures that cell growth and anabolic metabolism occur only under permissive conditions.

In skeletal muscle, appropriate mTORC1 activity is indispensable for growth, maintenance, and repair (12). Muscle-specific deletion of Raptor (*Rptor*), an essential subunit of mTORC1, results in profound atrophy and impaired force generation (13), highlighting the importance of proper mTORC1 activity in maintaining muscle homeostasis. On the contrary, sustained hyperactivation of mTORC1, whether induced experimentally through muscle-specific *Tsc1* deletion or arising naturally during aging, leads to progressive myopathy marked by fiber degeneration, metabolic stress, impaired proteostasis, and loss of regenerative capacity (8). In our previous work, we produced a mouse strain with depletion of both *Tsc1* and *Depdc5* in muscle tissue (*Ckm-Cre Tsc1^fl/fl^ Depdc5^fl/fl^*) to enforce constitutive mTORC1 pathway hyperactivation (6, 14). Unlike single-KO models (7, 8), which showed little pathology in young mice, *Ckm-Cre Tsc1^fl/fl^ Depdc5^fl/fl^* mice develop severe early-onset myopathy and skeletal muscle dysfunction. This phenotype was associated with excessive oxidative stress, impaired proteostasis, and accumulation of the autophagy adaptor SQSTM1/p62, resulting in dysregulation of myofiber homeostasis.

With recent advances in technologies, single-cell (sc) and single-nucleus (sn) RNA-Seq resources have mapped aging muscle across lifespan and diseases, providing valuable insights into cellular composition and aging-associated shifts in regenerative, immune, and fibrotic states (15–26). Spatial transcriptomic and imaging-based methods such as MERFISH (27) and smFISH (28) have further visualized the distribution of specific transcripts within intact myofibers and tissue sections, and multiplex proteomic platforms like CODEX (29) have profiled fiber-type mosaics at the protein level. Together, these approaches have greatly expanded our understanding of muscle biology and aging. However, they fall short in resolving the fiber-type complexity of myopathy; sc/snRNA-Seq relies on tissue dissociation and cannot resolve intact myofibers, and spatial methods to date have been constrained to targeted panels or low resolution, making it difficult to capture the full complexity of fiber-type heterogeneity in the myopathic process.

To directly address this gap, we applied Seq-Scope, a high-resolution spatial transcriptome profiling technology that we recently developed (30, 31). Its submicrometer resolution and unbiased whole-transcriptome coverage enable high-fidelity mapping of transcriptional diversity at the single-myofiber level. Using Seq-Scope, we profiled cross-sections of extensor digitorum longus (EDL) and soleus (SOL) muscles, two anatomically distinct tissues encompassing the major myofiber types, from *Ckm-Cre Tsc1^fl/fl^ Depdc5^fl/fl^* and control littermates. Our analyses revealed that mTORC1 hyperactivation elicits both global stress responses and fiber type–specific remodeling programs. Comparison between EDL and SOL demonstrated the striking context-dependent plasticity of MF2X, which engaged distinct signatures associated with metabolism regulation and cytoskeletal remodeling in each muscle. Within the same muscle, MF2B reprogrammed their classical glycolytic transcriptome, acquiring either MF2X-like oxidative signatures or undifferentiated myofiber-like developmental signatures. In addition, classical myopathic features, such as abnormal myofiber enlargement and basophilic fibers, were each linked to characteristic transcriptomic profiles with predicted functional outcomes. Overall, this study provides a histopathology-guided, single-fiber spatial framework for dissecting fiber type–specific remodeling under mTORC1 hyperactivation, providing molecular insight into the developmental processes of myopathies and a foundation for future mechanistic studies of muscle physiology and disease.

## Results

### Seq-Scope enables combined analysis of histopathology and single-myofiber transcriptomics.

We formerly generated a mouse model of unregulated mTORC1 hyperactivation by concomitantly deleting *Tsc1* and *Depdc5*, thereby achieving maximal activation of both Rheb and Rag arms of mTORC1 signaling (Figure 1A). mTORC1 hyperactivation in skeletal muscle severely disrupted tissue homeostasis, leading to loss of muscle function associated with various pathologies, including basophilic fibers, enlarged fibers, and centrally nucleated fibers (Figure 1A; white, black, and green arrows, respectively). To capture the molecular changes underlying these phenotypes at the resolution of individual myofibers, we used Seq-Scope, a spatial transcriptomics platform with submicrometer resolution (average 0.5–0.7 μm), which we recently developed and optimized (30, 31). By aggregating transcripts according to the histology-based myofiber segmentation, a spatial transcriptome could be precisely assigned into individual myofibers. This approach preserved fiber morphology and spatial context while revealing and mapping transcriptomic diversity.

In this study, we harvested EDL and SOL muscles from 4 *Ckm-Cre Tsc1^fl/fl^ Depdc5^fl/fl^* and 4 *Tsc1^fl/fl^* *Depdc5^fl/fl^* male littermates at 10 weeks of age. A total of 16 cross-sections (2 muscles per mouse) were placed onto Seq-Scope arrays for spatial transcriptomic profiling. The exact same sections were subjected to H&E and DAPI/wheat germ agglutinin (WGA) imaging on the array surface prior to transcript capture and library generation (Figure 1B; first and second row, respectively). Sequencing of the Seq-Scope library yielded a total of 700 million unique reads that successfully aligned to the mm10 reference genome with valid spatial barcodes. The resulting RNA density map (Figure 1B, third row) revealed strong localization of transcripts at the periphery of myofibers, consistent with known muscle architecture. Using WGA imaging data and the Cellpose segmentation algorithm to delineate cellular membranes, we segmented 8,616 individual myofibers across 16 tissue sections (Figure 1B, fourth row, and Supplemental Figure 1A; supplemental material available online with this article; https://doi.org/10.1172/jci.insight.203167DS1). Data from each sample were processed independently and then integrated and clustered to assign myofiber identities. The data showed minimal batch effects, aside from the expected separation of SOL and EDL with their distinct myofiber compositions (Figure 1C and Supplemental Figure 1, B and C). Myofibers were also clearly distinguished by the expression of their corresponding myosin heavy chain isoforms (Figure 1D and Supplemental Figure 1D) and by structural and metabolic genes associated with oxidative (MF1/MF2A) or glycolytic (MF2B) types (Supplemental Figure 1E).

To systematically assess the histopathological features observed in mutant muscle, we quantified the prevalence of centrally nucleated fibers, enlarged fibers, and basophilic fibers across all sections. Centrally nucleated fibers were identified by detecting DAPI-positive nuclei located within a segmented myofiber and not overlapping the WGA-defined sarcolemmal boundary (Figure 1E). Enlarged fibers were identified from the myofiber segmentation output by measuring the cross-sectional area of each fiber. Because fiber area can vary with sectioning angle, enlargement was defined using a section-specific internal reference: mutant fibers with cross-sectional area greater than the mean area of morphologically healthy fibers were classified as enlarged (Figure 1E and Supplemental Figure 1F).

Basophilia was handled differently because it represents a histological pattern, defined by enhanced hematoxylin accumulation in the cytoplasm, that could not be captured reliably by a single intensity threshold alone. We therefore defined 2 prespecified morphology-based categories and performed blinded manual annotation. Transitional basophilic fibers were defined by scattered intracytoplasmic dark hematoxylin-positive particles (Figure 1E), whereas developed basophilic fibers were defined by dense, stripe-like, or broadly filled hematoxylin-positive staining within the myofiber (Figure 1E). These criteria were provided to an independent rater blinded to genotype for manual labeling. The reproducibility of these annotations was further validated by interrater agreement across 4 independent raters, as well as by quantification of intrafiber hematoxylin signal (Supplemental Figure 1, G–I).

Basophilic fibers were observed only in mutant EDL muscle, while enlarged fibers were restricted to mutant SOL muscle (Figure 1F and Supplemental Figure 1J). Fibers with central nuclei were also more frequent in mutant SOL muscle. When stratified by fiber type, we found that MF2X fibers showed the highest frequency of pathological features relative to other fiber types, suggesting that MF2X fibers exhibit the strongest pathological response to mTORC1 hyperactivation in this dataset (Figure 1F and Supplemental Figure 1J).

### Identification of fiber type–specific responses to mTORC1 hyperactivation.

The intrinsic differences among skeletal muscle fiber types suggest that myofibers may exhibit distinct transcriptional responses to sustained mTORC1 hyperactivation (1). To examine this question, we conducted differential gene expression analysis comparing transcriptomic signatures between control and mutant muscle within each myofiber type, both at the fiber level (Figure 2A and Supplemental Figure 2A) and at the biological replicate level, using pseudobulk analysis (Supplemental Figure 3A). Although many genes were commonly upregulated in response to mTORC1 hyperactivation, a number of genes showed myofiber type–specific expression patterns (Figure 2B). Importantly, phospho-RPS6 immunostaining co-registered with fiber-type markers showed that mTORC1 activation was broadly increased across mutant fiber types and was not restricted to the fiber types with the most prominent pathological changes (Supplemental Figure 2B).

We classified genes upregulated under mTORC1 hyperactivation into global response genes (expressed across all 4 fiber types, 25%), partially specific response genes (expressed in 2 or 3 fiber types, 49.2% in total), and genes specific for MF2A (5.6%, 7 genes), MF2X (4.0%, 5 genes), and MF2B (16.1%, 20 genes) fiber types (Figure 2C and Supplemental Figure 2, C and D). Notably, the MF1 transcriptome showed only global responses, without unique MF1-specific signatures in this analysis. Despite some differences between analytical frameworks, similar fiber type–associated patterns were observed in the mouse-level pseudobulk analysis (Supplemental Figure 3, A–H).

To systematically evaluate the functional annotations associated with both global and fiber type–specific signatures, we performed gene ontology (GO) enrichment analysis. Globally expressed genes were enriched in pathways related to ion transport regulation, muscle differentiation, cytoskeletal and synaptic remodeling, protein localization to membranes, and proteostasis (Figure 2D). These pathways overlap with proteostasis- and remodeling-related programs observed in our previous bulk RNA-Seq findings (6), supporting the reproducibility of these transcriptional features across different platforms.

In contrast, the present study also identified fiber type–specific transcriptional patterns associated with mTORC1 hyperactivation (Figure 2, B and E). Gene ontology clustering showed that MF2B myofibers exhibited relatively limited enrichment of oxidative stress- or detoxification-related GO terms compared with other fiber types. Instead, MF2B-associated genes were enriched for carbohydrate metabolic pathways, including *Pkm*, *Phka1*, *Agl*, and *Pfkb3*, consistent with preservation of a glycolytic transcriptional profile under mTORC1 hyperactivation. By contrast, MF2A myofibers showed enrichment of genes associated with stress-response and protein quality-control pathways, including *Tfrc*, *Mt1*, *Psmd4*, and *Psmc3*, along with regulators such as *Igfbp5* and *Sln*. MF2X myofibers showed enrichment of remodeling-associated transcripts, including *Asb15*, *Sparcl1*, and *Actc1*, together with *Ctsb* and *Gpx3*, indicating a distinct structural and oxidative gene expression profile. Together, these results indicate an association between gene-level signatures and higher-order functional annotations (Figure 2F), supporting the conclusion that different fiber types exhibit distinct transcriptional programs associated with sustained mTORC1 hyperactivation.

### Distinct mTORC1-associated states of type IIx myofibers in different muscles.

MF2X represents the major fiber type consistently present in both the predominantly oxidative SOL muscle and the glycolytic EDL muscle, providing an opportunity to compare a shared fiber type across distinct muscle contexts (32). To investigate how MF2X fibers are altered under mTORC1 hyperactivation, we isolated a total of 1,447 MF2X myofibers from all tissue samples (Figure 3A, Supplemental Figure 1B, and Supplemental Figure 4A). Although control MF2X fibers from SOL and EDL were mixed in the UMAP manifold (Figure 3A), mTORC1-hyperactivated MF2X fibers separated into 2 distinct clusters corresponding to SOL- and EDL-derived MF2X fibers (Figure 3A). MF2X-SOL fibers showed strong enrichment for myofiber enlargement (Figure 3B), whereas MF2X-EDL fibers were preferentially associated with basophilia (Figure 3C and Supplemental Figure 1J).

Differential expression analysis comparing control and mutant MF2X-SOL or MF2X-EDL fibers revealed muscle context–associated transcriptional differences within the MF2X population (Supplemental Figure 4B). Among 216 genes highly upregulated under mTORC1 hyperactivation, 26 genes were classified as EDL specific and 72 genes were classified as SOL specific (Figure 3, D and E, and Supplemental Figure 4C). Gene score analysis showed that these SOL- and EDL-associated signatures were more prominent in mutant MF2X fibers than in control MF2X fibers (Figure 3F). Histology and spatial profiling further supported the separation between mutant SOL and EDL MF2X fibers at both the transcriptional and morphological levels (Figure 3G and Supplemental Figure 4, D and E).

We then performed gene set enrichment analysis using the SOL- and EDL-specific mTORC1-responsive genes (Figure 3H and Supplemental Figure 4F). Three categories of pathways emerged: those enriched in the global MF2X response (proteostasis and apoptosis, muscle contraction, myogenic development, and oxidative stress response), the SOL-specific MF2X response (cytoskeletal complex assembly and glucose homeostasis), and the EDL-specific MF2X response (ribonucleotide/purine metabolism and monosaccharide catabolism). Together, these findings indicate that, even within a shared fiber class, MF2X fibers exhibit muscle-specific transcriptional and histopathological programs under mTORC1 hyperactivation, supporting context-dependent divergence of type IIx myofibers.

### Type IIx myofibers in SOL undergo hypertrophy with structural remodeling.

Abnormal myofiber enlargement is a pathological hallmark observed in diverse muscle disorders and experimental models (1). Given that mTORC1 hyperactivation induced myofiber enlargement specifically in SOL muscles, we isolated and reanalyzed 2,972 SOL myofibers (Figure 4A). In mutant SOL muscles, enlarged fibers were mainly MF2X, and fiber-size analysis confirmed marked expansion of MF2X fibers compared with controls, whereas MF1 and most MF2A fibers were smaller (Figure 4, A–C, and Supplemental Figure 5A). These observations are consistent with IHC validation (Supplemental Figure 4E) and previously reported immunostaining quantification (8). We further noted a universal increase in *Myh1* expression and decrease in *Myh7* and *Myh2* expression (Supplemental Figure 5B). The proportion of MF2X fibers in general was elevated in mutant SOL muscle (Supplemental Figure 5C). Importantly, this shift was observed consistently across independent batches, suggesting a potential fiber-type shift under sustained mTORC1 hyperactivation.

In addition to exhibiting prominent hypertrophy, MF2X fibers also displayed more pronounced changes at the molecular level. Among the genes differentially expressed between control and mutant SOL tissues, MF2X fibers showed the strongest overall expression changes compared with other fiber types (Supplemental Figure 5D). We directly compared enlarged and nonenlarged fibers, enabling clearer identification of genes associated with mTORC1-driven hypertrophy (Figure 4D). These genes were generally expressed at low levels in non-hypertrophic fibers and showed strong positive correlations with myofiber size (Figure 4E and Supplemental Figure 5E).

Genes strongly correlated with fiber enlargement are classical autophagy regulators such as *Sqstm1* (p62), *Map1lc3a* (LC3A), and *Ubb*, as well as proteasome or protease components such as *Psmb5* and *Ctsd,* suggesting an association between myofiber hypertrophy and mTORC1 hyperactivation–driven proteostasis disruption on the transcriptional level. Genes involved in muscle growth and regeneration programs (e.g., *Igfbp5*, *Mustn1*, and *Ankrd1*) and structural genes associating with sarcomeric and cytoskeletal remodeling (e.g., *Actc1*, *Myl4*, *Mylpf*, *Actn3*, *Tpm1,* and *Mybpc3*) were also specifically upregulated in hypertrophic myofibers from mTORC1-hyperactivated muscles.

In addition, *Uchl1*, a ubiquitin C-terminal hydrolase linked to mTORC1 regulation, autophagy, and ubiquitin homeostasis in skeletal muscle (33, 34), was strongly correlated with fiber enlargement. Its elevated expression in enlarged fibers suggests a potential compensatory response to proteostasis disruption under sustained mTORC1 hyperactivation.

Importantly, the specific upregulation of *p62*, *Igfbp5*, *Actc1*, and *Uchl1* in hypertrophic myofibers was confirmed through IHC staining of their protein products (Figure 4F). This coordinated program, combining impaired proteostasis, persistent growth signaling, and cytoskeletal remodeling, was reflected in our GO network analysis (Figure 4G and Supplemental Figure 5F). Together, these transcriptional results suggested a potential connection of the molecular changes to the enlargement myopathy observed in SOL muscle (Figure 4H).

### Central nuclei are prevalent across various types of myofibers.

The presence of centrally located nuclei in skeletal myofibers is a well-recognized histopathological feature, commonly associated with regeneration, myopathies, and various muscle stress conditions (35, 36). Histological analysis revealed a higher proportion of centrally nucleated myofibers in mutant SOL compared with mutant EDL muscles (Figure 1F and Supplemental Figure 5G). Although no strong fiber-type specificity was detected, oxidative fibers had a subtle tendency to show more frequent central nuclei compared with glycolytic fibers (Supplemental Figure 5, G and H). To assess whether central nuclei regions display distinct transcriptional features, we segmented 430 central nuclei from 2 mutant EDL and 2 mutant SOL muscles using combined H&E, DAPI, and WGA staining. Nuclei were dilated by 2, 4, or 6 μm to capture sufficient transcripts, and their transcriptomes were compared with 24-μm hexagon profiles from the 4 selected sections to generate a spatial sn-like dataset (Supplemental Figure 5, I and J). The analysis did not reveal strong enrichment for a specific gene or pathway, likely reflecting that the central nuclei feature could happen for a variety of different myofibers without representing a specific pathway. However, we were able to identify several zinc finger proteins, such as *Zfp93* and *Zfp429*, which were substantially enriched in central nuclei regions and may be involved in positioning the nucleus during the injury (Supplemental Figure 5K).

### Basophilic fibers are associated with increased RNA and metabolism-related signatures.

Basophilic fibers represent a distinctive histological phenotype often associated with increased RNA content and muscle stress responses (37). Here, we isolated 48 developed basophilic fibers and 387 transitional basophilic fibers based on their histological patterns (Figure 5, A and B). Most basophilic fibers were restricted to MF2X fibers in mutant EDL muscle (Figure 5C and Supplemental Figure 6A). Consistent with the histological interpretation of basophilia, both transitional and developed basophilic fibers showed increased RNA content in the spatial transcriptomic dataset (Figure 5, D and E, and Supplemental Figure 6B), as well as increased RNA signal by RNA in situ hybridization (Supplemental Figure 6C).

Through differential expression analysis, we identified a set of transcripts enriched in basophilic fibers, including genes associated with lipid metabolism, mitochondrial function, oxidative metabolism, and nucleotide metabolism (Figure 5F). GO pathway enrichment analysis showed strong representation of pathways related to lipid metabolism, mitochondrial transport, the TCA cycle, electron transport, and nucleotide synthesis (Figure 5G and Supplemental Figure 6D). These findings identify metabolism-related transcriptional signatures associated with myofiber basophilia.

Consistent with this pattern, multiple fatty acid– and lipid metabolism–related genes, including *Fabp3*, *Acadl*, *Oxct1*, *Mfn2*, *Chpt1*, and *Gpx4*, were prominently enriched in basophilic fibers (Figure 5F). Basophilic fibers also showed elevated expression of oxidative metabolism–associated genes, including *Mb*, multiple *Cox* subunits, *Atp* genes, and several mitochondrially encoded transcripts. This oxidative signature was further supported by increased COX activity in basophilic regions of mutant EDL muscle (Supplemental Figure 6E).

Enrichment of nucleotide metabolism–associated pathways was also observed in basophilic fibers (Figure 5, F and G). Several TCA cycle–, redox-, and nucleotide metabolism–related genes, including *Idh2*, *Ogdh*, *Mdh1*, *Mdh2*, *Ldhb*, *Mpc2*, and *Nmrk2*, were elevated in this population. Together, these transcriptional signatures indicate that lipid-, oxidative-, and nucleotide metabolism–related gene expression is associated with the increased RNA content of basophilic fibers (Figure 5H).

To illustrate these associations within a histological context, *Fabp3*, *Mb*, and *Mdh1* were highlighted as representative genes associated with lipid handling, oxidative respiration, and nucleotide metabolism, respectively (Figure 5I, raw transcript view). In parallel, selected genes corresponding to these 3 gene sets were combined into cumulative scores, and their relative expression levels were assessed and visualized across the tissue (Figure 5I, Supplemental Figure 6A, and Supplemental Figure 6F). Taken together, our results indicate that basophilic MF2X fibers in mutant EDL muscle represent a distinct mTORC1-associated transcriptional state marked by increased RNA content and elevated expression of genes related to lipid, oxidative, and nucleotide metabolism.

### Type IIb fibers in EDL exhibit transcriptional heterogeneity under mTORC1 hyperactivation.

Type IIb fibers (MF2B), typically defined as the most glycolytic and fast-contracting population, have recently been shown to exhibit transcriptional variation under different contexts (38). Here, we identified transcriptional heterogeneity among MF2B fibers that was masked by aggressive batch correction during integration. This MF2B variation was initially observed in nonintegrated EDL clusters (Supplemental Figure 7, A–D). Since mutant samples showed greater variability across biological replicates, potentially reflecting biological heterogeneity at the 10-week time point, we first analyzed 2 mutant batches (KO-M1 and KO-M3) that integrated well even without batch correction (Figure 6, A and B). We then used these samples as reference datasets to define integration anchors and map all 4 mutant replicates into the same shared space (Supplemental Figure 8, A–C).

From these analyses, we identified 3 transcriptionally distinct MF2B subgroups under mTORC1 hyperactivation: MF2B subtype-1 (58% of mutant MF2B), which showed increased expression of transcripts resembling the shared mTORC1 hyperactivation signature; MF2B subtype-2 (22%), which showed increased expression of mitochondria-associated transcripts; and MF2B subtype-3 (20%), which was characterized by increased expression of developmental and remodeling-associated genes, including *Actc1* and *Myl4* (Figure 6C and Supplemental Figure 8D). These MF2B subgroups were represented across all 4 biological replicates (Supplemental Figure 7, D and E, and Supplemental Figure 8, E and F). All mutant subtypes exhibited elevated RNA content compared with controls, indicating altered transcriptional states under mTORC1 hyperactivation (Supplemental Figure 7F). Despite these transcriptomic differences, all MF2B subtypes maintained their fiber-type identity by robustly expressing the characteristic MF2B myosin isoform *Myh4* (Supplemental Figure 7B and Supplemental Figure 8G).

The elevated expression of mitochondria-associated transcripts, including *Atp* and *Cox* genes, in MF2B subtype-2 suggested a distinct mitochondrial transcript signature (Figure 6C). To quantify this feature, we generated a composite mitochondrial transcript score based on detectable mitochondrially encoded genes. This analysis showed that MF2B subtype-2 exhibited a relatively high mitochondrial transcript score, comparable to basophilic MF2X fibers within EDL muscle (Figure 6, C and D, Supplemental Figure 7, G and H, and Supplemental Figure 8H). However, unlike basophilic MF2X fibers, MF2B subtype-2 fibers largely maintained normal morphology (Figure 6, E and F). These findings indicate that a subset of MF2B fibers exhibits increased mitochondria-associated gene expression without altered myosin isoform identity or overt pathological morphology under mTORC1 hyperactivation.

MF2B subtype-3, marked by developmental and remodeling-associated transcripts, did not display obvious morphological phenotypes (Figure 6, E and G). Spatial transcript mapping and quantitative analyses supported the elevated expression of *Actc1* and *Myl4* in this subgroup (Figure 6G, Supplemental Figure 7I, and Supplemental Figure 8I). Co-immunostaining of ACTC1 and MYL4 revealed both overlapping and nonoverlapping fibers (Figure 6H), suggesting that this transcriptional state may involve multiple downstream regulatory patterns.

Together, these transcriptional findings indicate that type IIb fibers exhibit distinct transcriptional states under mTORC1 hyperactivation while maintaining their fiber-type identity (Figure 6I). These results highlight previously unrecognized heterogeneity within a fiber type that has traditionally been considered relatively uniform.

### mTORC1 hyperactivation drives macrophage-fibroblast accumulation in SOL muscle.

Aside from myofibers or myocytes, many non-myocytic cell types, including fibroblasts, endothelial cells, and immune cells, reside within skeletal muscle, where they provide essential support for tissue maintenance and homeostasis. To investigate how these populations are altered under mTORC1 hyperactivation, we profiled non-myocytes using 24-μm diameter, flat-to-flat width, hexagon spatial gridding. This analysis identified diverse cell types and tissue-associated structures, including macrophages, fibroblasts, neuromuscular junctions, and erythrocytes (RBCs) (Figure 7, A and B).

Resting non-myocytic cell types were broadly represented across batches (Supplemental Figure 9, A and B), but macrophage- and fibroblast-associated clusters were preferentially enriched in mutant SOL muscle (Figure 7C and Supplemental Figure 9C). The macrophage-associated cluster strongly expressed *Apoe*, consistent with a tissue-resident macrophage-like signature, whereas the activated fibroblast-associated cluster, separated from *Gsn*-expressing interstitial fibroblasts, expressed *Mgp*, *Thbs4*, *Col1a1*, and *Col3a1*, along with relatively low *Gsn* expression (Figure 7B and Supplemental Figure 9D). These observations suggest localized inflammatory and fibrotic remodeling in mutant SOL muscle, where histopathological features such as myofiber enlargement and central nuclei were also enriched. Macrophage- and fibroblast-associated clusters were frequently observed in close spatial proximity (Figure 7D). Consistent with this spatial transcriptomic pattern, immunofluorescence staining showed increased F4/80-positive macrophage signal and COL1A1-positive fibrotic signal in mutant SOL muscle, with enrichment in interstitial regions rather than diffuse distribution across myofibers (Supplemental Figure 9E). These findings support the presence of localized macrophage- and fibroblast-associated remodeling regions in mutant SOL muscle, consistent with prior studies showing that macrophage-fibroblast interactions can contribute to fibrotic tissue remodeling (39–41).

Overall, these results show that mTORC1 hyperactivation in myofibers is accompanied by a muscle context–dependent non-myocyte remodeling program. The preferential enrichment of macrophage- and fibroblast-associated clusters in mutant SOL, together with their close spatial proximity to each other and to regions of myofiber pathology, supports the presence of localized inflammatory and fibrotic remodeling in this muscle. Because the analysis was performed using 24-μm spatial hexagon bins at a single time point, these data do not establish the temporal order or causal direction between myofiber pathology and non-myocyte remodeling. Nevertheless, they identify a spatially organized macrophage-fibroblast–associated tissue state that is selectively linked to the mutant SOL microenvironment under sustained mTORC1 hyperactivation.

## Discussion

Histopathological features such as basophilic fibers, fiber enlargement, and central nuclei have long served as hallmarks of skeletal muscle disease (37, 42, 43), yet the molecular programs underlying these morphologies remain poorly understood. Traditional approaches have struggled to connect phenotype with transcriptional state at the level of single fibers, and scRNA- and snRNA-Seq lose their associated histological information (15–26). By combining high-resolution spatial transcriptomics with image-guided fiber segmentation, we establish a framework to directly link morphological phenotypes with their transcriptomes. This pathology-guided, single-fiber analysis reveals how sustained mTORC1 hyperactivation drives diverse and fiber type–specific pathological responses across distinct muscle groups.

Even though the difference in susceptibility between EDL and SOL has long been recognized (44–46), our study is among the first, to our knowledge, to show that pathology-induced transcriptomic outcomes diverge sharply between these two muscles. In SOL, MF2X fibers underwent abnormal enlargement, whereas in EDL, MF2X fibers developed a basophilic phenotype. These contrasting trajectories indicate that MF2X fibers do not adopt a uniform pathological program but instead respond in a muscle-dependent manner. Given the distinct baseline fiber-type compositions and metabolic environments of SOL and EDL, it is plausible that local tissue context modulates how MF2X fibers respond to mTORC1 hyperactivation. In particular, the continuous activation and loading of SOL may contribute to its growth phenotype, whereas the relatively low usage of EDL might favor alternative pathological outcomes. It is also possible that MF2X fibers arising during development contain latent differences in identity, even if their baseline transcriptomes appear highly similar, which could contribute to muscle-specific outcomes under stress. Because mTORC1 hyperactivation is induced early in this model, the present study cannot determine whether these divergent MF2X outcomes arise from developmental differences, local muscle environment, postnatal pathological remodeling, or a combination of these factors. Thus, we interpret the SOL- and EDL-associated MF2X phenotypes as muscle context–associated pathological states rather than as evidence for a single defined developmental or environmental mechanism.

Abnormally enlarged fibers were identified to engage sustained growth signaling, disrupted autophagic homeostasis, and structural remodeling, which is in line with the result from a previous study using bulk tissues (8). These processes were also shown to drive physiological myofiber hypertrophy in the context of mechanical overload (47). However, under continuous mTORC1 activation, they may tip the balance away from normal growth toward pathological remodeling. Even though hyperactive mTORC1 drives ongoing anabolic activity, including increased protein synthesis and ribosomal biogenesis, it simultaneously suppresses autophagy, thereby compromising proteostasis. This impaired capacity to clear damaged proteins and organelles likely exacerbates the imbalance between growth and quality control. Alongside these defects, cytoskeletal remodeling signatures, including reorganization of actin filaments and contractile apparatus components, suggest structural adaptation aimed at accommodating increased fiber size. Together, these processes may contribute to hypertrophic pathologies, but future time-course or perturbation studies will be required to determine causal order.

In EDL, MF2X fibers predominantly developed the basophilic phenotype, marked by increased RNA content and gene expression patterns related to lipid metabolism, oxidative metabolism, and nucleotide synthesis. Previous studies have linked basophilia to ribonucleic acid accumulation and regenerative or metabolically oxidative muscle states (37), and our oligo(dT) and COX validations support increased poly(A) RNA signal and oxidative histochemical activity in these fibers. However, the current data do not measure metabolic flux and therefore cannot determine whether lipid-supported respiration directly fuels nucleotide synthesis or RNA accumulation. Under mTORC1 hyperactivation, where anabolic programs strongly promote RNA and protein synthesis, these lipid-, oxidative-, and nucleotide-associated signals may reflect a coordinated basophilia-associated program rather than a proven metabolic route. Alternative explanations, including mTORC1-driven ribosome biogenesis, regeneration-like programs, altered mitochondrial content, or local tissue state, remain possible. In either case, the phenomenon underscores the transcriptional plasticity of MF2X fibers, which can express oxidative gene signatures within an otherwise glycolytic muscle environment.

In addition to these pathological features, our analysis also uncovered unexpected adaptability within MF2B fibers (Figure 6I). Traditionally viewed as the most glycolytic and least flexible fiber type (48, 49), MF2B in EDL diverged into 3 distinct subgroups under mTORC1 hyperactivation. One subgroup displayed the canonical stress-response program, consistent with the global transcriptomic changes induced by mTORC1 activation. The second subgroup, however, showed elevated expression of mitochondrial and oxidative genes while retaining a morphologically normal appearance. We speculate that this reflects a compensatory shift, in which a fraction of MF2B fibers adopt oxidative features to support aerobic metabolism that would normally be contributed by MF2X fibers, which are diverted into the basophilic trajectory. The third subgroup re-expressed developmental and structural regulators such as *Actc1* and *Myl4*, pointing to a remodeling program that may reflect partial dedifferentiation or activation of regenerative pathways. These divergent outcomes suggest that mTORC1 hyperactivation does not impose a uniform response in glycolytic fibers, but instead interacts with local or intrinsic cues to channel MF2B into distinct transcriptional trajectories. With responses largely independent from overt morphological pathologies, we speculate that these heterogeneous programs may be shaped and affected by surrounding non-myocytes or neighboring fibers. Importantly, despite these wide variations in transcriptome response, they maintained their MF2B identity by expressing their characteristic myosin isoform *Myh4* at robust levels. These findings reveal that MF2B fibers, long considered metabolically rigid, can mount heterogeneous transcriptional responses when challenged, suggesting a previously underappreciated capacity for remodeling within this fiber type.

We also observed variability in phenotype prevalence across muscles and biological replicates. This variability may reflect differences in disease stage, local tissue context, sectioning, or biological heterogeneity at the 10-week time point. Although all sections were included in the integrative analysis, selected well-aligned samples were used for initial discovery of fine-scale heterogeneity, followed by validation across all replicates using reference-based integration and module scoring.

A limitation of our study is that it was performed in a murine model of sustained mTORC1 hyperactivation, and further work will be needed to determine whether similar adaptive programs operate in other pathological contexts or human diseases. In addition, a direct cell-resolved comparison with skeletal muscle–specific *Tsc1* single-KO muscle is currently limited by the lack of a closely matched sc/snRNA-Seq or high-resolution spatial transcriptomic dataset. Therefore, the present study should be interpreted primarily as a single-fiber spatial characterization of the *Tsc1/Depdc5* double-KO model, rather than as a formal comparative analysis between *Tsc1* single-KO and *Tsc1/Depdc5* double-KO transcriptomes. In this context, *Tsc1* single-KO and *Tsc1/Depdc5* double-KO models should be viewed as complementary rather than interchangeable. *Tsc1* single-KO models may be better suited for studying gradual aging-associated mTORC1 pathology, whereas the *Tsc1/Depdc5* double-KO model provides a severe early-onset setting that facilitates high-resolution analysis of histopathological and fiber type–specific transcriptional programs.

In conclusion, our study provides a comprehensive view of how sustained mTORC1 hyperactivation reshapes skeletal muscle at single-fiber resolution. By integrating pathology-guided segmentation with spatial transcriptomics, we uncovered fiber type–specific adaptations, including divergent MF2X trajectories and unexpected heterogeneity within MF2B, as well as non-myocytic responses associated with fibrotic remodeling. These findings extend our understanding of the molecular programs that underlie classic histopathological features of myopathy and emphasize the plasticity of fiber types under sustained mTORC1 hyperactivation. Overall, this work highlights the value of spatially resolved transcriptomics for linking morphology to molecular state and establishes a framework for future investigations into the mechanisms that govern muscle pathology.

## Methods

### Sex as a biological variable.

In this study, spatial transcriptomic analyses were performed in male mice. Our previous characterization of the same mouse model showed that mTORC1-driven myopathy and associated cardiac and respiratory defects occur in both male and female mice (6, 14), indicating that the core pathological consequences of mTORC1 hyperactivation are not sex restricted. Many functional and molecular assays in that work were carried out in males, and the present study builds on that dataset by adding high-resolution, fiber type–specific, and muscle-specific transcriptional information in the same sex. Consequently, the spatial and single-myofiber programs described here are defined in male skeletal muscle, and it remains unknown whether the detailed histopathological and transcriptional trajectories are identical in females. Future studies that include both sexes and are prospectively powered for sex-stratified analyses will be required to determine whether sex modifies susceptibility to mTORC1 hyperactivation, the responses of individual fiber types, or associated non-myocytic remodeling.

### Rodent muscular mTORC1 hyperactivation model.

As previously described (6, 14), *Tsc1^fl/fl^* (stock 005680) and *Ckm-Cre* (stock 006475) mice were obtained from The Jackson Laboratory, and *Depdc5^fl/fl^* mice were obtained from the European Mouse Mutant Archive (EM:10459). *Tsc1^fl/fl^* *Depdc5^fl/fl^* double-KO mice were generated by interbreeding, and progeny were subsequently crossed with *Ckm-Cre* mice to produce skeletal muscle–specific KO animals. All mice were maintained under protocols approved by the University of Michigan IACUC. To minimize batch effects, 4 control and 4 KO littermates were selected for tissue harvest. Immediately after dissection, muscle tissues were embedded in OCT compound (23-730-571, Thermo Fisher Scientific) and snap-frozen in liquid nitrogen–cooled 2-methylbutane (MX0760, Sigma-Aldrich).

### Seq-Scope array production (1st-Seq).

Seq-Scope procedures were described in detail previously (30, 31). Seq-Scope is a solid-phase transcriptome spatial capture system that has undergone several upgrades. The latest version was built on the Illumina NovaSeq 6000 platform with a 7 mm × 7 mm imaging area. Barcoded capture probe clusters on the array surface were generated using HDMI32-DraI, a custom single-stranded oligonucleotide library (IDT), and Read1-DraI sequencing primer through a sequence-by-synthesis strategy. The sequencing output FASTQ file generated during array construction contained the barcode sequences and their corresponding *x-y* coordinates.

The array, originating from an Illumina NovaSeq 6000 flow cell, contains 4 channels with 7 mm × 70 mm imaging areas. Arrays were split and diced into suitable sizes for storage and experimental use. Before application, arrays required several preprocessing steps. Arrays were first washed 3 times with nuclease-free water, followed by overnight incubation at 37°C with a DraI (R0129, NEB)/CIAP (M0525, NEB) enzyme mixture. The following day, arrays were treated with exonuclease I (M2903, NEB) for 45 minutes at 37 °C, and then washed sequentially: 3 times with nuclease-free water, 3 times with 0.1 N NaOH for 5 minutes each, and 3 times with 0.1 M Tris (pH 7.5).

### Seq-Scope spatial transcriptome library generation and sequencing (2nd-Seq).

Seq-Scope library generation was performed according to our previous paper (30). OCT-mounted frozen muscle blocks stored at –80°C were equilibrated in a cryostat (Leica CM3050S) at –15°C for 1 hour prior to sectioning. Sections were cut at 10-μm thickness with a 5° cutting angle and placed onto cold Seq-Scope arrays, and then warmed to room temperature to promote tight attachment. Tissue fixation was performed on the array with 4% formaldehyde (15170, Electron Microscopy Sciences) at room temperature for 10 minutes, followed by 3 PBS washes. Sections were then costained with DAPI (Invitrogen, D21490) and Alexa Fluor-488 WGA (Invitrogen, W11261) for 15 minutes at room temperature, washed 3 times with PBS, mounted in 5% glycerol, and imaged on a Keyence digital darkroom system for whole-tissue DAPI/WGA capture. The coverslip was then gently removed to avoid tissue damage, after which H&E staining was performed and whole-tissue H&E images were captured with a Keyence microscope.

Tissue permeabilization was carried out by incubating sections with 0.2 U/μL collagenase I (17018-029, Thermo Fisher Scientific) at 37°C for 20 minutes, followed by 1 mg/mL pepsin (P7000, Sigma-Aldrich) in 0.1 M HCl at 37°C for 10 minutes. Reverse transcription (RT) was performed between the permeabilized tissue and array surface using an RT mixture (1× RT buffer, EP0751, Thermo Fisher Scientific; 4% Ficoll PM-400, F4375-10G, Sigma-Aldrich; 1 mM dNTPs, N0477L, NEB; RNase inhibitor, 30281, Lucigen; Maxima H-RTase, EP0751, Thermo Fisher Scientific) and incubated overnight at 42°C in a humidified chamber. The following day, unbound single-stranded DNA probes were digested with exonuclease I (M2903, NEB). Tissue was removed by incubation with a digestion cocktail (100 mM Tris, pH 8.0; 100 mM NaCl; 2% SDS; 5 mM EDTA; 16 U/mL Proteinase K, P8107S, NEB) at 37°C for 40 minutes. Arrays were then washed 3 times each with nuclease-free water, 0.1 N NaOH (5 min per wash), and 0.1 M Tris, pH 7.5.

Library generation was performed immediately after washing. Arrays were incubated for 2 hours at 37°C in a humidified chamber with a library synthesis mixture (1× NEBuffer-2, NEB; 10 μM TruSeq Read2-conjugated random primer, IDT; 1 mM dNTPs, N0477, NEB; Klenow Fragment, M0212, NEB; nuclease-free water). The arrays were then washed with nuclease-free water, and libraries were collected twice by 0.1 N NaOH elution (5 min each). Eluates were neutralized with 3 M potassium acetate, pH 5.5, and purified with AMPure XP beads (1.2× bead/sample ratio, A63881, Beckman Coulter) according to the manufacturer’s instructions.

Libraries were amplified in 2 rounds of PCR using Kapa HiFi HotStart ReadyMix (KK2602, KAPA Biosystems). The first round used 2 μM TruSeq forward and reverse primers as described previously (30, 31), and products were purified with AMPure XP beads (1× bead/sample ratio). In the second round, TruSeq indexing primers were applied, and products were size-selected with AMPure XP beads (0.6× bead/sample ratio). Library quality was assessed on an Agilent 2100 Bioanalyzer, with additional purification performed if necessary. Final libraries were sequenced using paired-end 100-cycle reads.

### Raw data processing.

Seq-Scope data processing was performed using the NovaScope pipeline, as described in detail previously (30). A 1st-Seq data table was generated from the 1st-Seq FASTQ files, containing array region lookup indices, quality-filtered barcode sequences, and corresponding spatial coordinates. The 2nd-Seq FASTQ reads were then processed against this table by (a) assigning array indices through region lookup, (b) mapping reads to the spatial barcode map using spatial barcode sequences (HDMI), (c) filtering barcoded reads with validated barcode sequences, and (d) assigning spatial coordinates to the identified reads. Reads with assigned spatial information were aligned to the genome using STAR (50), and a spatial digital gene expression (sDGE) matrix was generated.

### Histology and sDGE alignment.

H&E images were aligned to sDGE matrix using the *historef* package (version 0.1.3), which detects fiducial marks visible in both modalities to guide image registration, as described previously (30). DAPI/WGA images were subsequently aligned to the processed H&E images using the Georeferencer function in QGIS (version 3.22.9).

### Myofiber segmentation and hexagon gridding.

Myofiber segmentation was performed in Cellpose (51) using WGA-stained myofiber membranes as input. A custom segmentation model was trained to optimize fiber identification, and manual adjustments were applied when necessary. Segmentation masks generated by Cellpose were exported as NumPy arrays and processed with custom Python scripts to label and isolate individual fibers. Transcripts within each segmented myofiber were aggregated and stored as an aggregated spatial digital gene expression matrix. Longitudinal and oblique myofibers, which are minimally represented within the cross-sections, were ruled out during pixel size counting.

For non-myocyte analysis, spatial hexagon grids (24 μm, nonoverlapping) were generated using NovaScope, where transcripts within each grid were aggregated and treated as a single unit, with spatial coordinates assigned to the hexagon center.

### Data analysis and visualization.

Single-myofiber datasets were analyzed using the Seurat package (52, 53). Myofibers with low feature counts (nFeature cutoff: 200) were removed to optimize clustering performance. Each batch was normalized with SCTransform, and datasets were integrated using SCT-normalized values with either internal anchors or reference-based anchors. Principal components were calculated using the RunPCA function, and high-quality components were selected to generate UMAP embeddings, followed by cluster identification with the FindNeighbors and FindClusters functions. Myofiber clusters were annotated based on marker gene expression, and unique transcript signatures were identified with the FindMarkers and FindAllMarkers functions.

Gene ontology enrichment analysis was performed with ClusterProfiler (54) using the org.Mm.eg.db annotation (Bioconductor project). Data visualization was supported by various R packages including Seurat, ggplot2, ggvenn (version 0.1.10), ggVennDiagram (55), ComplexUpset (version 1.3.3), ComplexHeatmap (56), and enrichplot (54). Cumulative gene scores were calculated based on the expression level of indicated gene sets across all fiber segments. Spatial myofiber projections were generated from segmentation NumPy arrays using custom python scripts. Raw spatial gene expression was visualized with a custom software platform that displayed both histology and the sDGE matrix.

Hexagon-based spatial transcriptomes (24 μm nonoverlapping grids) were analyzed independently without dataset integration, primarily to detect non-myocytes. Each hexagon was treated as a single cell. Normalization and clustering were performed in Seurat using SCTransform, RunPCA, and RunUMAP functions, followed by marker-based annotation.

### Pseudobulk RNA-Seq analysis.

For replicate-aware differential expression analysis, raw transcript counts from individual myofibers were aggregated by mouse, muscle, and fiber type, generating pseudobulk samples for each mouse × muscle × fiber-type category. Differential expression was performed with DESeq2 by comparing control and mutant groups within each muscle fiber-type group. Interaction models were additionally used to identify fiber type–specific transcriptional responses to *Tsc1/Depdc5* deletion.

### IHC.

Frozen muscle sections were equilibrated to room temperature before processing. Sections were permeabilized with 0.5% Triton X-100 in PBS for 5 minutes, followed by 3 PBS washes (5 min each). Fiber-type immunostaining was performed using a Mouse on Mouse (M.O.M.) Immunodetection kit (Vector Laboratories, BMK-2202) according to the manufacturer’s instructions. Briefly, sections were incubated overnight at 4°C with M.O.M. Mouse Ig Blocking reagent, then with primary antibodies diluted in M.O.M. diluent at optimized concentrations. Alexa Fluor–conjugated secondary antibodies (Invitrogen) were diluted in M.O.M. diluent and applied for 60 minutes at room temperature. For membrane visualization, sections were incubated with Alexa Fluor 488–conjugated WGA (Invitrogen, W11261) for 10 minutes prior to mounting with ProLong Gold Antifade Mountant (Invitrogen, P36934). Images were acquired using a Zeiss LSM 980 confocal microscope equipped with an Airyscan 2 detector. Primary antibodies myosin heavy chain type I (BA-D5), myosin heavy chain type IIA (SC-71), and myosin heavy chain type IIB (BF-F3) were acquired from Developmental Studies Hybridoma Bank, University of Iowa. ACTC1 (66125-1-IG) and MYL4 (67533-1-IG) were purchased from ProteinTech. UCHL1 (PA5-29012), IGFBP5 (PA5-37369), and F4/80 (14-4801-82) were purchased from Invitrogen. SQSTM1/p62 (catalog 5114), COL1A1 (catalog 72026), and Phospho-S6 Ribosomal Protein (Ser235/236) (catalog 2211) were purchased from Cell Signaling Technology. The following Alexa Fluor–conjugated secondary antibodies were obtained from Invitrogen: goat anti-mouse IgM heavy chain Alexa Fluor 647 (A-21238), goat anti-mouse IgG2b cross-adsorbed Alexa Fluor 555 (A-21147), F(ab)-goat anti-mouse IgG1 Fc Alexa Fluor 405 (A66789), goat anti-mouse IgG2b cross-adsorbed Alexa Fluor 350 (A-21140), goat anti-mouse IgG1 cross-adsorbed Alexa Fluor 555 (A-21127), donkey anti-mouse IgG (H+L) highly cross-adsorbed Alexa Fluor 594 (A-21203), donkey anti-mouse IgG (H+L) highly cross-adsorbed Alexa Fluor 647 (A-31571), donkey anti-rabbit IgG (H+L) highly cross-adsorbed Alexa Fluor 647 (A-31573), donkey anti-rabbit IgG (H+L) highly cross-adsorbed Alexa Fluor 594 (A-21207), and donkey anti-rat IgG (H+L) highly cross-adsorbed Alexa Fluor 594 (A-21209).

### Cytochrome c oxidase staining.

Cytochrome c oxidase A solution was prepared in PBS containing 0.75 g sucrose and 0.05 g DAB (Sigma-Aldrich, D5637), adjusted to pH 7.6. Cytochrome c oxidase B solution was prepared in PBS containing 0.001 g catalase and 0.05 g cytochrome c, adjusted to pH 7.6. Cytochrome c oxidase A and B were mixed to generate the incubation medium and applied to frozen sections. Slides were incubated for 1 hour at room temperature, rinsed briefly in running distilled water, dehydrated through graded ethanol, cleared in Safeclear, and mounted with Permount mounting medium (Electron Microscopy Sciences, 17986-01). Cytochrome c oxidase intensity was quantified using ImageJ (NIH).

### Oligo-dT fluorescence in situ hybridization.

Frozen tissue sections were fixed in 4% formaldehyde for 10 minutes, washed 3 times with PBS, and permeabilized in 100% methanol at −20°C for 20 minutes. Sections were washed 3 times with 2× SSC buffer (Sigma-Aldrich, SRE0068) and incubated in encoding wash buffer containing 30% formamide in 2× SSC for 10 minutes. Hybridization buffer containing 2× SSC, 30% formamide (Invitrogen, AM9342), yeast tRNA (Thermo Fisher Scientific, 15401029), 10% dextran sulfate (Thermo Fisher Scientific, J63606.14), RNase inhibitor, and 2 μM fluorophore–conjugated oligo-dT probe (IDT, TTTTTTTTTTTTTTT-3’ ATTO 590) was applied to the tissue and incubated in a humidified chamber at 37°C for 48 hours protected from light. Slides were then washed 3 times with 2× SSC, stained with 5 μg/mL Alexa Fluor 488–conjugated WGA for 10 minutes, washed again, mounted in 85% glycerol, and imaged.

### Statistics.

Genes significantly upregulated in mutant muscle compared with control muscle were first filtered using stringent cutoffs on expression level and fold-change, followed by systematic classification on fiber types using a combination of Kruskal-Wallis and pairwise Wilcoxon’s tests. The Kruskal-Wallis test identified genes with significantly higher expression in their top-expressing fiber type, thereby defining fiber type–specific signatures (adjusted *P* value < 0.05). Pairwise Wilcoxon’s tests were subsequently conducted to compare expression levels across fiber types, allowing us to define partially shared gene groups as well as globally enriched transcripts (adjusted *P* value < 0.05). The Wald test was used in the pseudobulk analysis to identify differentially expressed genes. A 1-sided Fisher’s exact test was used in enrichment analyses to identify significantly enriched pathways. *P* values of less than 0.05 were considered significant.

### Study approval.

All animal procedures were approved by the University of Michigan IACUC and performed in accordance with institutional guidelines.

### Data availability.

All source data, source code for custom analyses reported in the paper, and selected processed datasets are deposited in the Deep Blue repository (https://doi.org/10.7302/p25z-mk61), where the spatial barcode-coordinate structure required for reuse of the Seq-Scope data is preserved. Next-generation library sequencing data (2nd-Seq) are also publicly available in NCBI’s Gene Expression Omnibus (GEO GSE338936). Supporting data values for the main and Supplemental figures are provided in the Supporting Data Values Excel file.

## Author contributions

JEH designed research studies, conducted experiments, acquired data, analyzed data, and wrote the manuscript. QZ and WC analyzed data. HMK and SVB designed research studies and wrote the manuscript. MK designed research studies, conducted experiments, analyzed data, and wrote the manuscript. JHL designed research studies, analyzed data, and wrote the manuscript.

## Conflict of interest

JHL is an inventor on intellectual property related to Seq-Scope and has a potential financial interest through the University of Michigan in connection with Salus Biomed Co.

## Funding support

This work is the result of NIH funding, in whole or in part, and is subject to the NIH Public Access Policy. Through acceptance of this federal funding, the NIH has been given a right to make the work publicly available in PubMed Central.

- Taubman Institute Innovation Projects (to HMK and JHL).
- NIH grants R01AG079163 (to MK and JHL), U01HL137182 (to HMK), UG3CA268091 and UH3CA268091 (to JHL), R01AG086251 (to SVB), P30AG024824, P30AG013283, P30DK034933, P30DK089503, P30CA046592, P30AR069620, and U2CDK110768.
- Taiwanese Government Fellowship (to JEH).
- Chan Zuckerberg Initiative (to HMK).
- Glenn Foundation Core Grants (to SVB and JHL).

## Supplementary Material

Supplemental data

Supporting data values

## Figures and Tables

**Figure 1:**
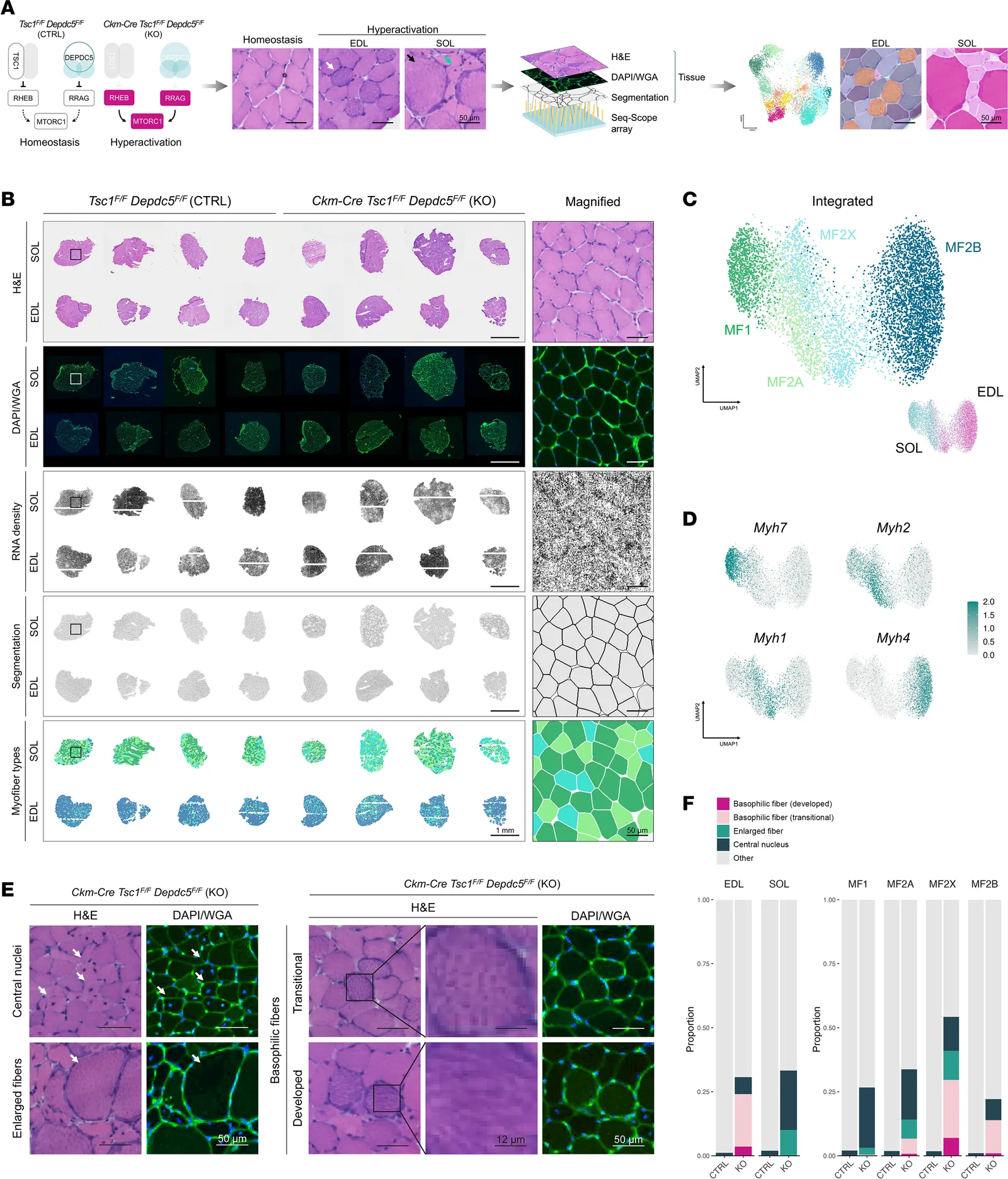
Seq-Scope enables histopathology-guided single-myofiber transcriptome analysis. (**A**) Schematic of the experimental workflow. Arrows indicate pathological phenotypes including basophilia (white), hypertrophy (black), and central nuclei (green). H&E histology, DAPI/WGA imaging, and Seq-Scope whole transcriptome analysis were performed on the same slide, which allowed precise segmentation and single-myofiber transcriptome analysis. Scale bar: 50 μm. (**B**) Cross-sections of control and mutant skeletal muscles examined by Seq-Scope. From top to bottom, images are displayed as H&E histology, DAPI/WGA staining, RNA density map from Seq-Scope transcriptome data, single-myofiber segmentation, and spatial myofiber type projection, according to the clusters identified in **C**. *Tsc1^fl/fl^ Depdc5^fl/fl^*: control; *Ckm-Cre Tsc1^fl/fl^ Depdc5^fl/fl^*: mutant. MF1: myofiber type I; MF2A: myofiber type IIa; MF2X: myofiber type IIx; MF2B: myofiber type IIb. Scale bars: 1 mm and 50 μm. (**C**) UMAP manifolds displaying the major myofiber types identified by multidimensional clustering. Datasets were integrated across mice. Each point (*n* = 8,616) represents an individual myofiber and is colored according to its corresponding myofiber type (center) or its originating muscle depot (lower right). (**D**) UMAP manifold colored by the expression levels of myosin heavy chain (*Myh*) isoforms that represent each myofiber type. (**E**) Pathological phenotypes observed in mTORC1 hyperactivated skeletal muscle. Left panel: centrally nucleated myofibers (arrows in the top row) and hypertrophic fibers (arrows in the bottom row). Right panel: transitional basophilic fibers (top row, scattered dark particles) and developed basophilic fibers (bottom row, dense and strip-like filling). Boxed areas are magnified on the right. Scale bars: 12 μm and 50 μm. (**F**) Quantification of pathological phenotype prevalence by muscle depot (left) or by myofiber type (right). “Other” includes myofibers from control muscles as well as morphologically normal fibers from mutant muscles. CTRL, control; KO, mutant.

**Figure 2:**
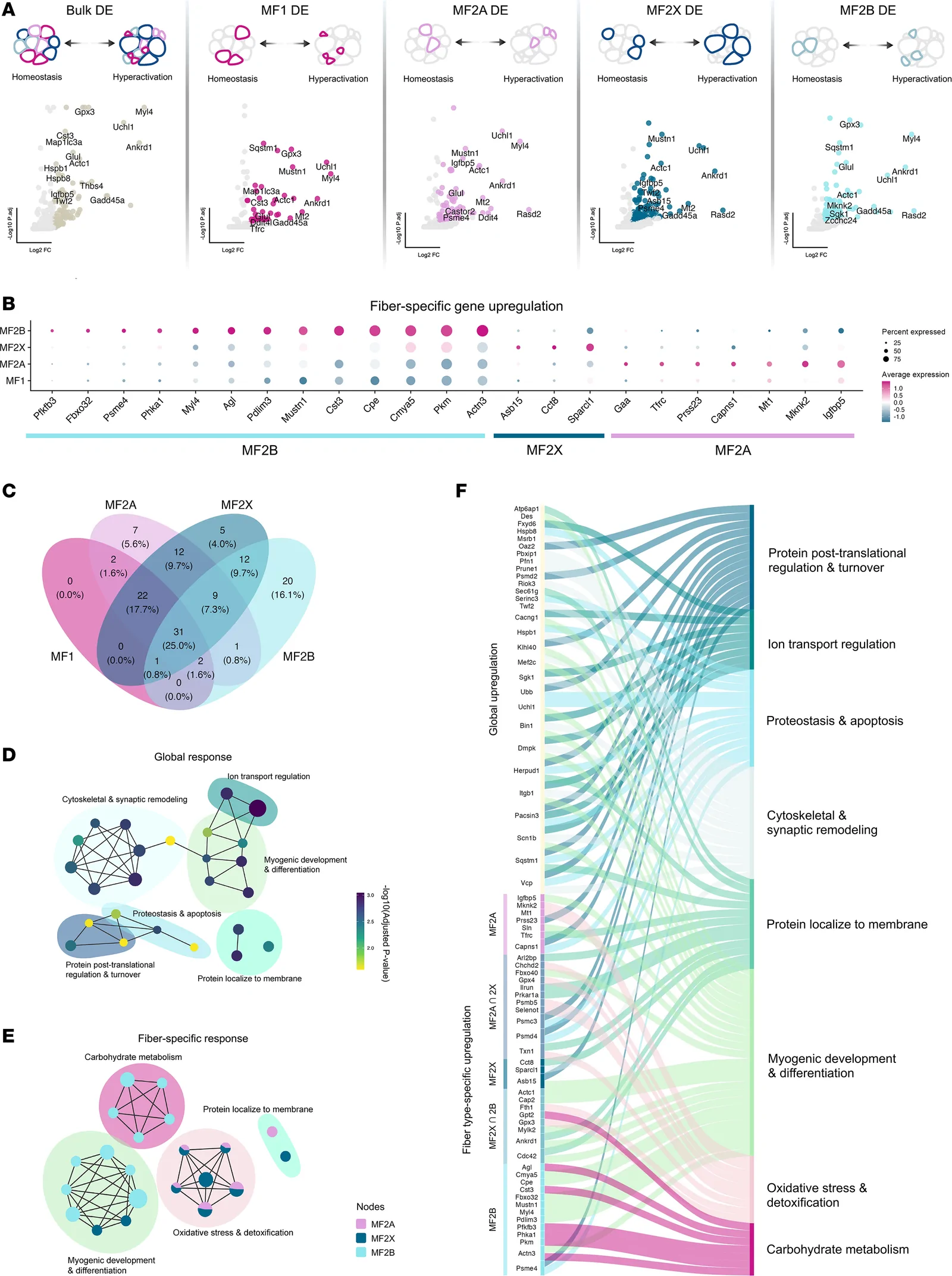
Identification of fiber type–specific responses to mTORC1 hyperactivation. (**A**) Schematic of the analytical workflow for fiber type–specific differential expression (DE) analysis comparing control (homeostasis) and mutant (hyperactivation) muscle. Upregulated genes determined by Wald’s test (adjusted *P* value < 0.05, log_2_FC > 1.5). (**B**) Representative expression patterns of genes that are exclusively upregulated in individual myofiber types, selected from a broader set (Supplemental Figure 2C). Exclusivity was determined by the Kruskal-Wallis test (adjusted *P* value < 0.05). Exclusively expressed genes were not identified in mutant MF1 fibers. (**C**) Venn diagram depicting the uniqueness and overlap of upregulated genes across different myofiber types, with numbers and percentages shown for both shared and unique gene sets. (**D**) Genes upregulated across all 4 fiber types (global response) were analyzed by gene ontology (GO) enrichment and visualized as a network. Each node represents a GO term, grouped by functional proximity within the enrichment hierarchy. Node size indicates the number of genes associated with the term, and node color reflects enrichment significance determined by 1-sided Fisher’s exact test (adjusted *P* value). (**E**) Genes selectively upregulated in specific fiber types were analyzed using compareGO. GO terms were grouped by functional proximity. Node size reflects the number of contributing genes, and node colors are shown as pie charts indicating the relative enrichment strength determined by 1-sided Fisher’s exact test (adjusted *P* value) contributed by each fiber type. (**F**) Genes globally upregulated across all fiber types and genes selectively upregulated in specific fiber types were mapped to enriched GO groups. The origin of each gene set is indicated by the stratum, and connecting threads represent gene-to-pathway relationships, colored by GO term identity.

**Figure 3:**
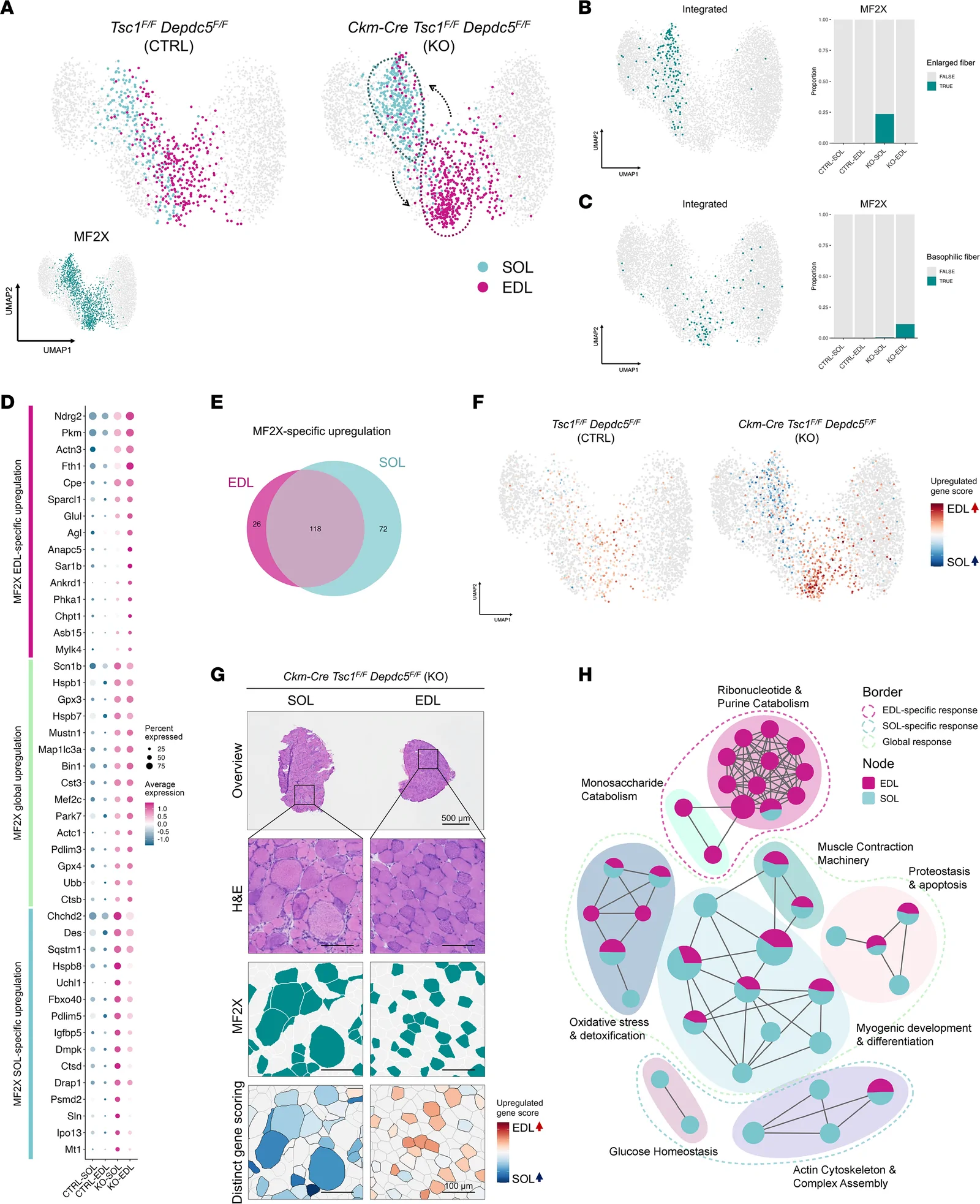
Distinct hyperactive mTORC1 responses of type IIx myofibers in different muscles. (**A**) UMAP visualization of myofiber data points separated as control (left) or mutant (right) groups, with muscle identity (EDL: red; SOL: blue) annotated within the original MF2X depot (lower left). Dotted arrows and shapes indicate the bidirectional shift of the MF2X subclusters in response to mTORC1 hyperactivation. (**B** and **C**) Enlarged fibers (**B**) and developed basophilic fibers (**C**) marked in the UMAP manifold (left). Proportions quantified from MF2X cluster of indicated muscles and presented in a bar graph (right). (**D**) Representative expression patterns of genes upregulated in response to mTORC1 hyperactivation in all MF2X fibers (MF2X global upregulation) or specifically in MF2X of EDL or MF2X of SOL, selected from a broader set in Supplemental Figure 4C. Exclusivity was determined using pairwise Wilcoxon’s tests (adjusted *P* value < 0.05). (**E**) Venn diagram depicting the uniqueness and overlap of genes upregulated in MF2X of SOL and MF2X of EDL. (**F**) Cumulative module score of muscle-specific upregulated genes displayed in integrated UMAP manifold with control (left) and mutant (right) myofiber data points separated. (**G**) Spatial images of representative mutant sections from each muscle. Boxed areas are magnified below (H&E). MF2X myofiber identity and cumulative module scores representing the axis of SOL- and EDL-specific responses are visualized. Scale bars: 100 μm and 500 μm. (**H**) GO enrichment network representing group of genes upregulated in response to mTORC1 hyperactivation in all MF2X fibers (global response) or specifically in MF2X of EDL (EDL-specific) or MF2X of SOL (SOL-specific). Pathways are grouped by functional similarity. Node size reflects the number of genes associated with each pathway, and node colors are shown as pie charts indicating the relative enrichment significance determined by 1-sided Fisher’s exact test (adjusted *P* values) contributed by each muscle-specific gene set. For the complete results with GO term names, see Supplemental Figure 4F.

**Figure 4:**
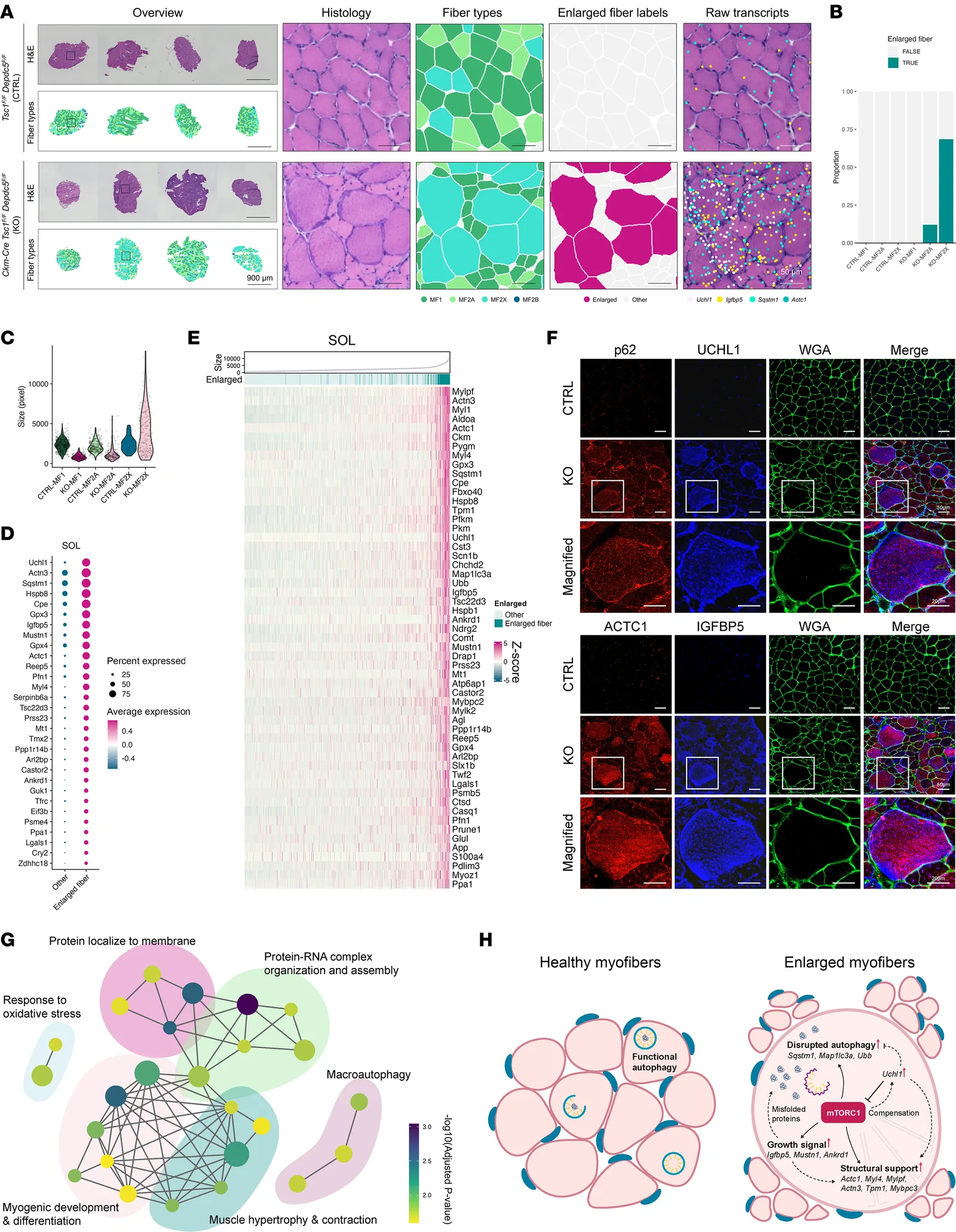
Type IIx myofibers in SOL undergo hypertrophy with structural remodeling. (**A**) Spatial images of SOL sections. Left: histology overview of 4 control sections (top) and 4 mutant sections (bottom) overlaid with fiber type images. Right: spatial images of magnified boxed area from corresponding overview, presented from left to right as histology, fiber types, enlarged fiber labels, and raw transcripts plotted as colored dots. Scale bars: 900 μm and 50 μm. (**B**) Prevalence of enlarged fibers in different myofiber clusters of SOL quantified and presented as a bar graph. (**C**) Size of fibers in different myofiber clusters of SOL calculated and presented as a violin plot. One pixel corresponds to ~0.57 μm^2^. (**D**) Expression of genes highly upregulated in enlarged fibers presented in a dot plot. (**E**) Correlation between fiber size and gene expression. Myofibers (*x* axis) are ordered from small to large. Positively correlated genes (*y* axis) are ranked by correlation strength. The expression level of the genes is colored based on *z* scores. RNA counts within a single fiber were normalized by fiber size to estimate RNA density. (**F**) Immunostaining of indicated proteins in control or mutant SOL muscle. WGA staining (green) outlines myofiber borders. Staining was performed on 2 serial sections (top and bottom). Boxed areas highlighting an enlarged fiber are magnified at the bottom. Representative images are shown from a single staining experiment. Scale bars: 20 μm and 50 μm. (**G**) GO enrichment network based on genes upregulated in enlarged fibers. Pathways grouped by functional similarity. Node size corresponds to the number of genes associated with each pathway, and node color reflects enrichment significance determined by one-sided Fisher’s exact test (adjusted *P* value). For the complete results with GO term names, see Supplemental Figure 5F. (**H**) Schematic diagram illustrating transcriptional signatures associated with pathological hypertrophy in mTORC1-activated type IIx myofibers in SOL.

**Figure 5:**
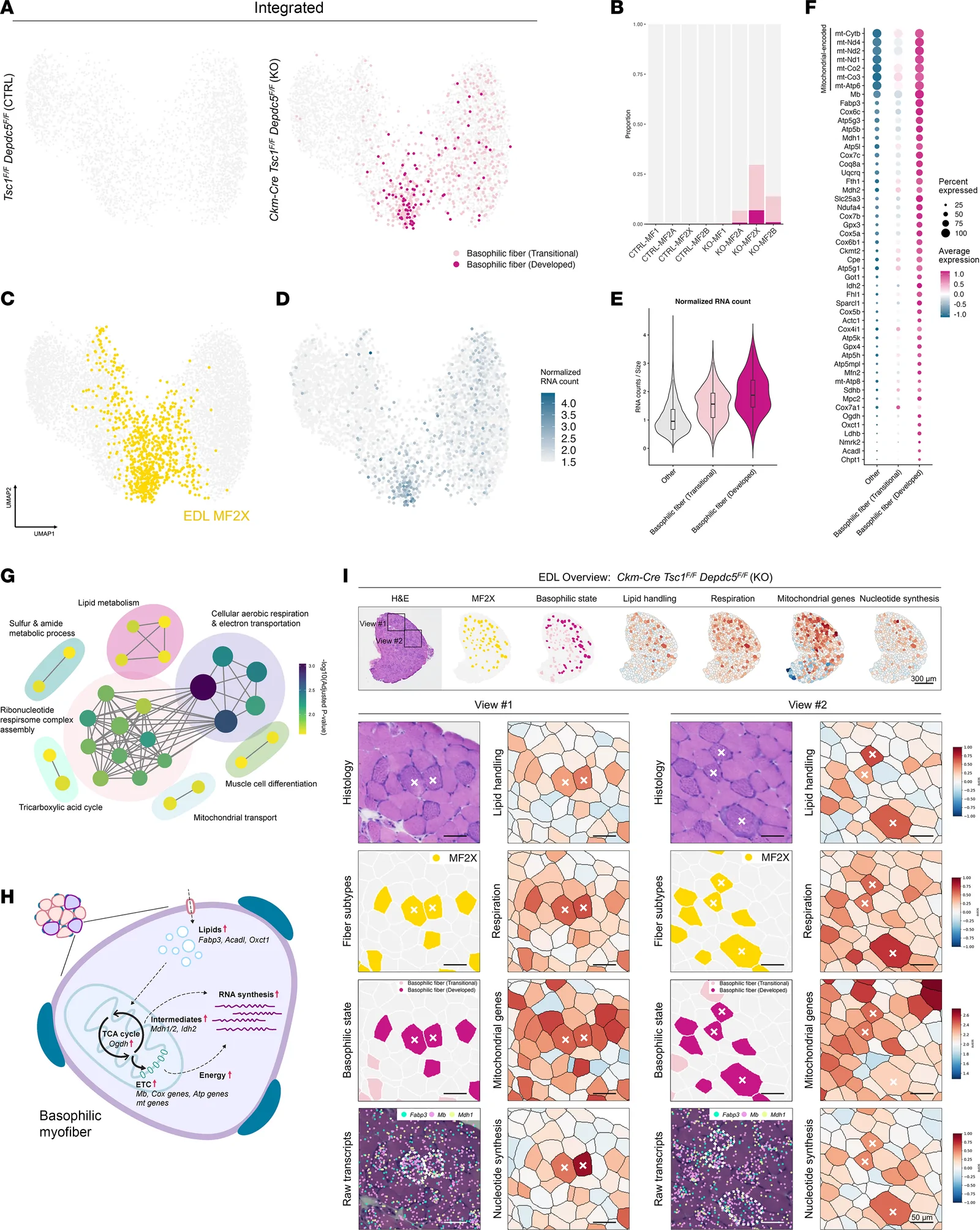
Basophilic fibers are associated with increased RNA and metabolism-related transcriptional signatures. (**A**) Integrated UMAP manifold, colored with transitional (pink) and developed (red) basophilic fibers, shown separately for control (left) and mutant (right) groups. (**B**) The prevalence of basophilia was quantified for each fiber-type cluster. (**C**) UMAP manifold displaying MF2X myofibers originating from EDL muscles (yellow). (**D**) UMAP manifold displaying RNA density (size-normalized RNA count) of each myofiber. (**E**) Violin plot visualizing the distribution of size-normalized RNA counts for transitional (pink) and developed (red) basophilic fibers as well as other fibers (gray). (**F**) Dot plot visualizing the expression of genes upregulated in basophilic fibers. (**G**) GO enrichment network analysis based on genes specifically upregulated in basophilic fibers. Pathways grouped by functional similarity. Node size corresponds to the number of genes associated with each pathway, and node color reflects enrichment significance determined by 1-sided Fisher’s exact test (adjusted *P* value). For the complete results with GO term names, see Supplemental Figure 6D. (**H**) Schematic diagram illustrating transcriptional signatures associated with mTORC1-induced basophilia in type IIx fibers in EDL. (**I**) Spatial images of a representative section from mutant EDL. Top: overview images presented from left to right as H&E histology, MF2X identity, basophilic states, and cumulative score maps for the following genes: lipid handling score (*Fabp3,* *Acadl*, *Oxct1*, *Mfn2*, *Chpt1*, *Gpx4*, and *Ckmt2*); respiration score (*Mb*, *Cox* genes, and *Atp* genes, see **F**); mitochondrial gene score (mitochondrial genome-encoded genes, see **F**); nucleotide synthesis score (*Idh2*, *Ogdh*, *Mdh1, Mdh2*, *Ldhb*, *Mpc2*, and *Nmrk2*). Bottom*:* representative basophilic fibers magnified in 2 views, corresponding to the boxes above in the H&E image. Raw transcripts are shown in colored dots as indicated in insets. Crosses and dotted borders highlight representative MF2X fibers. Scale bars: 300 μm and 50 μm. For the images from other mutant EDL sections, see Supplemental Figure 6A.

**Figure 6:**
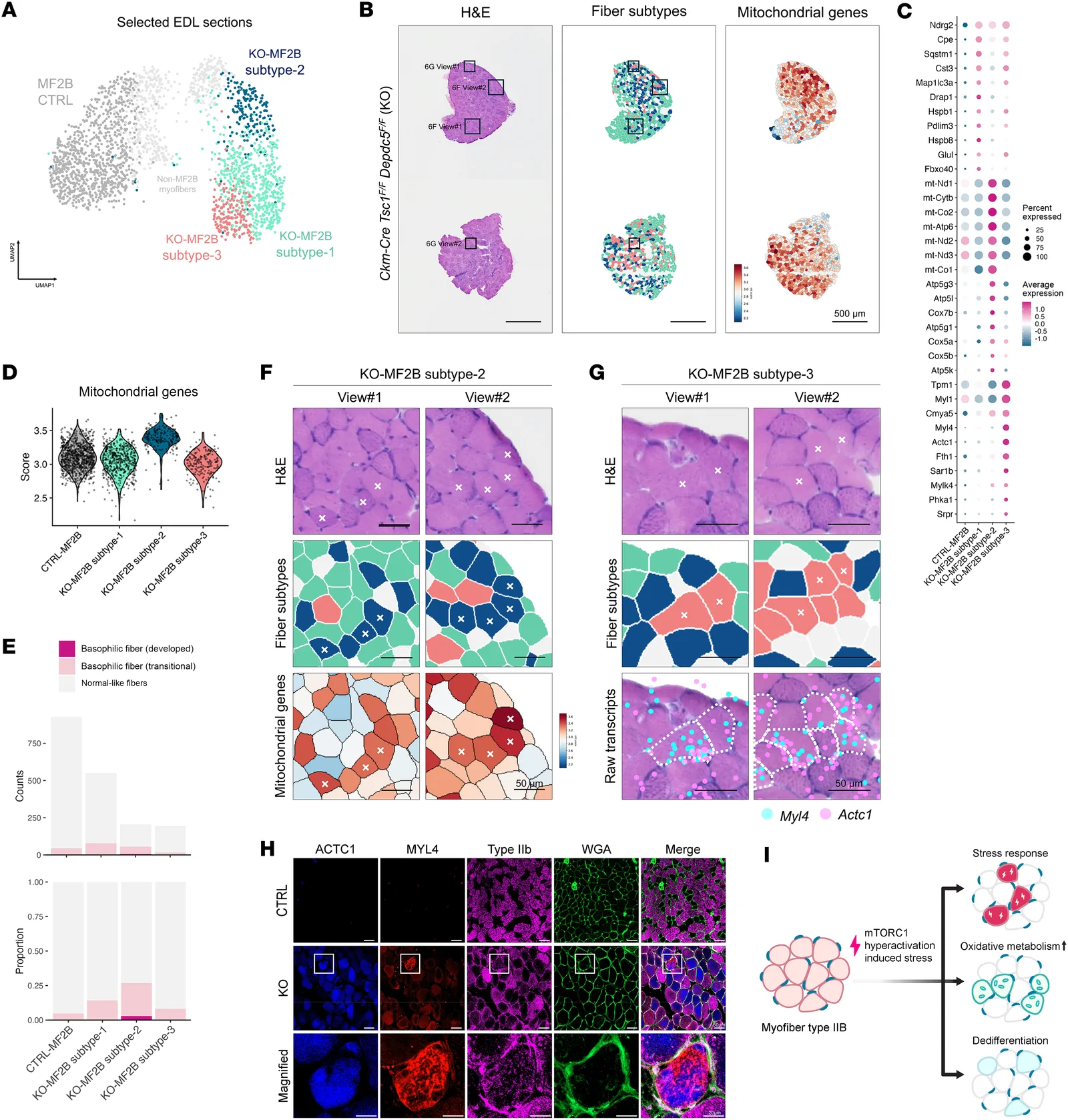
Type IIb fibers in EDL exhibit 3 distinct transcriptional states without gross morphological alterations. Myofibers from EDL muscles were subset and analyzed to reveal MF2B heterogeneity. (**A**) UMAP manifold showed MF2B clusters in selected control and mutant EDL sections (*n* = 2). Control MF2B cluster and non-MF2B clusters are labeled in dark gray and light gray, respectively. For full analysis, see Supplemental Figures 7 and 8. (**B**) Spatial images of selected mutant EDL sections. Left: H&E histology. Middle: MF2B fiber subtype map, according to the clustering presented in **A**. Right: Spatial map of a cumulative score created with the expression level of mitochondrial genome-encoded genes. Boxed areas in H&E images are magnified in **F** and **G**. Scale bar: 500 μm. (**C**) Expression of genes upregulated in each KO-MF2B subtype. (**D**) Mitochondrial gene cumulative score compared between control and mutant MF2B subtypes. (**E**) Frequency of basophilic fibers in control and mutant MF2B subtypes. (**F**) Representative views of MF2B subtype-2 with histology (H&E), fiber-subtype map, and mitochondrial gene cumulative score map. Crosses indicate representative subtype-2 myofibers. Scale bar: 50 μm. (**G**) Representative views of MF2B subtype-3 with histology (H&E), fiber-subtype map, and raw transcript plots of indicated genes. Crosses and dotted borders highlight representative subtype-3 myofibers. Scale bar: 50 μm.(**H**) Immunostaining of indicated proteins in control and mutant EDL muscles. WGA staining (green) delineates myofiber membranes. Boxed areas are magnified at the bottom. Representative images are shown from a single staining experiment. Scale bars: 20 μm and 50 μm. (**I**) Schematic diagram illustrating differential transcriptional states of MF2B fibers under sustained mTORC1 hyperactivation.

**Figure 7:**
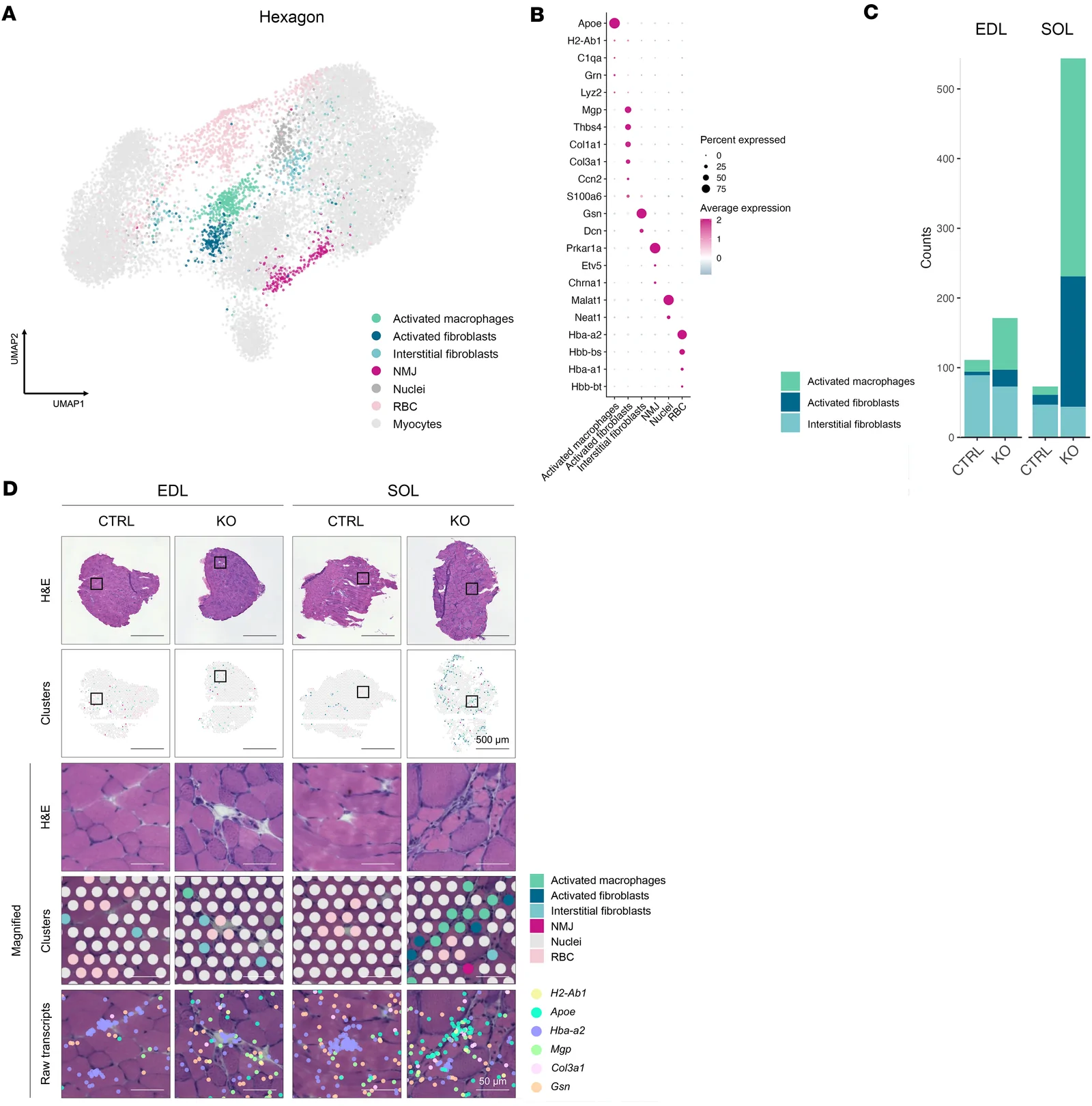
mTORC1 hyperactivation drives macrophage-fibroblast accumulation in SOL muscle. Non-myocytes were identified in skeletal muscle using 24 μm nonoverlapping hexagon gridding. (**A**) UMAP manifold of the hexagonal grid dataset displaying non-myocyte clusters, conducted without batch correction. (**B**) Expression of non-myocyte markers in different cell types. (**C**) Quantification of macrophage and fibroblast cell-type prevalence in control and mutant mice. (**D**) Spatial images showing H&E histology and cluster maps in representative muscle sections from each group. Boxed areas are magnified. Transcript expressions are displayed as colored dots as indicated Scale bars: 50 μm and 500 μm.

## Version 1 08/20/2026

In-Press Preview

## Version 2 10/08/2026

Electronic publication
