# Supplementary material for: Histopathology and spatial transcriptomics jointly map myofiber-specific pathological programs in mTORC1-driven myopathy
Source: JCI Insight. 2026 Aug 20;11(19):e203167. doi: 10.1172/jci.insight.203167 (PMC13726413; doi:10.1172/jci.insight.203167)
Supplement: Supplementary file 1 [file jciinsight-11-203167-s240.pdf]

Supplemental Figure Legends

Supplemental Figure 1. Stratification of single-myofiber transcriptome into classical myofiber types.

(**A**) Distribution of unique RNA features (number of detected genes; left) and transcript counts (right) per segmented myofiber, shown separately for EDL and SOL. A minimum cutoff of 200 unique RNA features was applied prior to integration and downstream analyses.

(**B**) Data integration performance. The UMAP manifold is colored according to the conditions (left) and batches (right) of individual myofibers. All eight biological replicates, from either control or mutant mice, contributed to all clusters with similar proportions.

(**C**) Proportion of myofiber types across muscles (left) and batches (right).

(**D**) Expression of myofiber-specific myosin heavy chain isoforms across designated myofiber types.

(**E**) Dot plot representing the expression of fiber type-specific marker genes in each myofiber type cluster. Dot size represents the proportion of myofibers expressing each gene, and dot color represents the average expression level.

(**F**) Myofiber size distribution across groups.

(**G**) Distribution of average hematoxylin-derived signal per segmented myofiber across basophilic groups.

(**H–I**) Fleiss' kappa values for developed basophilia and transitional basophilia across four independent raters. Error bars indicate 95% confidence intervals (**H**). Heatmaps showing pairwise Cohen's kappa values (left) and pairwise percent agreement (right) among four independent raters for transitional basophilia (top) and developed basophilia (bottom) (**I**).

(**J**) Quantification of pathology prevalence across muscle and fiber type. Sample points represent biological replicates. Box plots indicate median and interquartile range. Whiskers indicate data range. Asterisks indicate statistically significant differences by two-sided Mann-Whitney U test (**P* value < 0.05).

Supplemental Figure 2. Validation of fiber type-specific and global gene expression comparisons.

(**A**) From the UMAP manifold showing fiber type identities (Figure 1C), two condition-specific manifolds were generated. Dotted double-arrow lines indicate the fiber type-matched comparisons used for differential expression analysis.

(**B**) Spatial validation of mTORC1 activation across myofiber types in mutant soleus and EDL through phospho-RPS6 (p-RPS6) immunostaining. Fiber types were assigned using an adjacent section stained for myosin isoforms: type I, red; type IIa, blue; type IIb, green; and type IIx, black. Merged images show p-RPS6 signal overlaid with WGA, and the fiber-type panels indicate the corresponding fiber-type identities. p-RPS6 intensity at the single-fiber level, stratified by muscle and fiber type, was quantified and tested using Wilcoxon test ($n = 7$). Asterisks indicate statistical significance as shown (**P* value < 0.05, ***P* value < 0.01, ****P* value < 0.001).

(**C**) Expression of mTORC1 hyperactivation-induced genes that are upregulated only in specific myofiber types in mutant muscle. The intersection symbol (∩) indicates genes that are upregulated in both of the indicated myofiber types.

(**D**) Expression of globally upregulated genes induced by mTORC1 hyperactivation across all myofiber types.

Supplemental Figure 3. Pseudobulk differential expression analysis by muscle and fiber type

(**A**) Volcano plots showing replicate-aware differential expression between mutant and control myofibers using DESeq2 after pseudobulk aggregation by mouse × muscle × fiber type. Each point represents a gene. Significantly upregulated and downregulated genes are determined by the Wald test, highlighted, and representative genes are labeled.

(**B**) Genes identified by DESeq2 interaction analysis as exhibiting fiber type-specific upregulation in mutant muscle. Shown are significantly upregulated genes determined by the Wald test in KO samples (adjusted *P* value < 0.05) whose responses differed by fiber type. Genes previously identified in the individual fiber-level analysis are highlighted in cyan. MF1 in EDL and MF2B in SOL were excluded because these fiber types were only minimally represented in the corresponding muscles.

(**C–H**) Box plots showing expression levels of all genes shown in (**B**). Each panel shows one gene, with expression grouped by genotype for the indicated muscle and fiber type. Expression values were derived from pseudobulk samples aggregated by mouse × muscle × fiber type. Individual points represent biological replicates.

Supplemental Figure 4. Validation of MF2X fiber responses under mTORC1 hyperactivation

(**A**) Contribution of different experimental batches to each indicated dataset of MF2X myofibers.

(**B**) Differential expression analyses of MF2X fibers in SOL (left) or EDL (right) between control and mutant mice. Significantly upregulated or downregulated genes (adjusted *P* value < 0.05, $|Log_2FC| > 1.5$; highlighted) are determined by the Wald test.

(**C**) Expression of the complete list of genes that are upregulated in response to mTORC1 hyperactivation in all MF2X fibers (MF2X global upregulation) or specifically in MF2X of EDL or MF2X of SOL. Exclusivity was determined using pairwise Wilcoxon tests (adjusted *P* value < 0.05).

(**D**) Spatial images of muscle-specific MF2X cumulative module scores (MF2X distinct gene scoring) representing the axis of SOL- and EDL-specific responses.

(**E**) Immunostaining of indicated fiber types in control or mutant EDL or SOL muscle. Type I (red), IIa (blue) and IIb (green) fibers are visualized through specific antibody staining while type IIx fibers appear unstained (black). WGA staining (white) outlines myofiber membranes and extracellular matrix. Boxed areas in Merge column are magnified at right. Representative images are shown from a single staining experiment.

(**F**) Heatmap showing the result of gene set enrichment analysis of genes upregulated in response to mTORC1 hyperactivation in all MF2X fibers (MF2X global upregulation) or specifically in MF2X of EDL or MF2X of SOL. Color represents the enrichment significance determined by one-sided Fisher's Exact test (adjusted *P* values). The result was used to construct the GO enrichment network shown in Figure 3H.

Supplemental Figure 5. Transcriptomic characteristics of pathological features including myofiber enlargement and central nuclei.

(**A**) Myofiber size visualized in integrated UMAP space, shown separately for control (left) and mutant (right) groups.

(**B**) Expression of myofiber-specific myosin heavy chain isoforms across control and mutant SOL muscle batches.

(**C**) Proportion of myofiber types in control and mutant SOL muscle batches.

(**D**) Expression of mTORC1 hyperactivation-driven upregulated genes in SOL myofiber types. Most of these genes show highest expression in MF2X fibers in mutant mice.

(**E**) RNA count-fiber size correlation. Left: correlation between transcript counts per myofiber (RNA Counts) and fiber size. Right: correlation between size-normalized transcript density (RNA Counts / myofiber) and fiber size (Cell size).

(**F**) GO enrichment analysis of genes upregulated in enlarged fibers, corresponding to the GO network shown in Figure 4G.

(**G**) Centrally nucleated myofibers are marked in integrated UMAP space, separated by control (left) and mutant (right) groups.

(**H**) Proportion of centrally nucleated myofibers in indicated subsets.

(**I**) Histology-based spatial single-nuclei-like analysis. Central nuclei were identified from the histology images, and central nuclear transcriptome was extracted from spatial transcriptome with different radial ranges, reflecting lateral diffusion of transcripts. Analysis compared whole-tissue transcriptomes (segmented by 24 μm hexagon gridding) with central-nuclei transcriptomes.

(**J**) Identification of centrally nucleated fibers by combining DAPI/WGA and H&E staining. Central nuclei with different radial ranges are visualized in a gradient on top of H&E images. Boxed areas are magnified below.

(**K**) Comparison of central-nuclei transcriptomes with whole-tissue transcriptomes. Differentially expressed genes between tissue transcriptome and central nuclei transcriptomes of different radial ranges were shown in volcano plots. Upregulated (red) and downregulated genes (green) ($|Log_2FC| > 1.5$) are highlighted; significantly upregulated genes (adjusted *P* value < 0.05) are determined by the Wald test and are labeled.

Supplemental Figure 6. Basophilic fibers in EDL show increased RNA content and oxidative metabolism–related transcriptional signatures

(**A**) Spatial images of mutant EDL sections (full set). Left panel from top to bottom: H&E histology, basophilia map, and MF2X cluster map. Right panel from top to bottom: maps of lipid-handling module score, respiration module score, mitochondrial gene module score, and nucleotide synthesis module score, calculated as in Figure 5I.

(**B**) Distribution of RNA counts and fiber sizes across basophilic fiber states.

(**C**) Spatial validation of increased RNA content in basophilic fibers of mTORC1-activated muscle using Oligo-dT fluorescence in situ hybridization. Boxed regions in mutant sections are shown at higher magnification. The intensity of mRNA signal was quantified and compared between normal fibers (Other) and basophilic fibers using Wilcoxon test (n = 7). Error bars indicate 95% confidence intervals. Asterisks indicate statistical significance as shown (**P* value < 0.05, ***P* value < 0.01, ****P* value < 0.001).

(**D**) Enriched GO terms corresponding to nodes in the basophilic fiber functional network (Figure 5G).

(**E**) Histological validation of basophilic MF2X fibers in *Tsc1/Depdc5* double-knockout EDL muscle. The intensity of COX staining was quantified and compared between healthy-like fibers

(Other) and basophilic fibers using the Wilcoxon test (n = 7). Error bars indicate 95% confidence intervals. Asterisks indicate statistical significance as shown (**P* value < 0.05, ***P* value < 0.01, ****P* value < 0.001).

(**F**) Distribution of cumulative module scores across basophilic fiber states.

Supplemental Figure 7. Diversification of MF2B responses to mTORC1 hyperactivation.

(**A**) UMAP manifold displaying myofiber types from all EDL sections, constructed without batch correction.

(**B**) Expression of myofiber-specific myosin heavy chain isoforms across designated myofiber types. All MF2B myofiber subtypes express *Myh4*, a key marker of MF2B.

(**C**) UMAP manifold displaying experimental batches from all EDL sections, constructed without batch correction.

(**D**) Proportion of individual batches in different myofiber types, presented in a bar graph.

(**E**) Spatial images of 8 EDL sections (4 control and 4 mutant) used for the analyses in (**A–D**). From top to bottom, H&E histology, fiber type map, mitochondrial gene cumulative score, and *Myl4*+*Actc1* combined score maps are shown.

(**F**) Distribution of size-normalized RNA counts in different MF2B subclusters.

(**G**) Mitochondrial gene cumulative score in different myofiber clusters of EDL.

(**H**) Expression of mitochondrial genome-encoded genes in different myofiber clusters of EDL.

(**I**) Expression of indicated genes in mutant MF2B subtype-3 in EDL.

Supplemental Figure 8. MF2B subtypes were observed across all four mutant EDL replicates through Seurat reference-based anchor integration

(**A–B**) UMAPs show the identified MF2B subtype structure (**A**) and the contribution of each biological replicate (**B**).

(**C**) Spatial maps show the distribution of MF2B subtypes in each mutant EDL section.

(**D**) Expression level of representative marker genes across each MF2B subtype and MF2X.

(**E–F**) Box and stacked bar plots summarize sample count (top) and composition (bottom) across subtypes.

(**G–I**) violin plots show subtype-associated transcriptome signatures and mitochondrial gene score.

Supplemental Figure 9. Hexagonal grid analyses of non-myocyte populations.

(**A**) UMAP manifold, colored with batches (left), conditions (middle), or muscle (right), constructed without batch correction. Non-myocytes are highlighted in indicated colors, while myocytes are greyed out.

(**B**) Distribution of unique transcript counts (left) and gene numbers (RNA features, right) per non-myocytes of indicated batches.

(**C**) Quantification of macrophage and fibroblast cell type prevalence in indicated batches.

(**D**) Expression of fibroblast activity-associated genes across non-myocyte clusters.

(**E**) Histological validation of inflammatory and fibrotic remodeling in mTORC1-activated soleus muscle. Control and mutant tissues were stained for F4/80 (red), COL1A1 (green), and WGA (white). Representative images are shown from a single staining experiment.

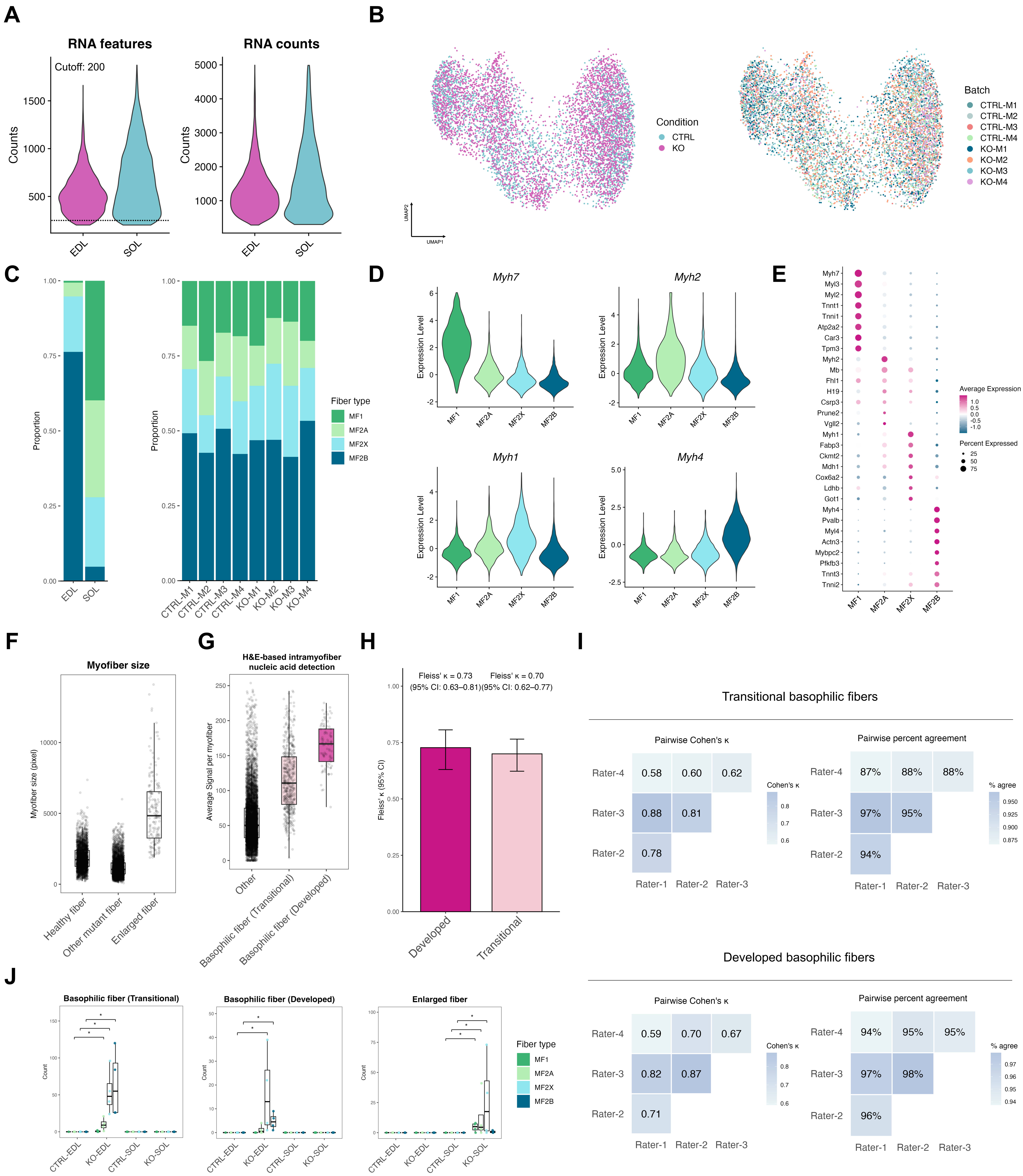
A
RNA features
Cutoff: 200
RNA counts
Counts
EDL
SOL
B
Condition
CTRL
KO
Batch
CTRL-M1
CTRL-M2
CTRL-M3
CTRL-M4
KO-M1
KO-M2
KO-M3
KO-M4
UMAP2
UMAP1
C
Proportion
Fiber type
MF1
MF2A
MF2X
MF2B
D
Myh7
Myh2
Myh1
Myh4
Expression Level
E
Average Expression
Percent Expressed
F
Myofiber size
Myofiber size (pixel)
Healthy fiber
Other mutant fiber
Enlarged fiber
G
H&E-based intramyofiber nucleic acid detection
Average Signal per myofiber
Other
Basophilic fiber (Transitional)
Basophilic fiber (Developed)
H
Fleiss' κ = 0.73
(95% CI: 0.63–0.81)
Fleiss' κ = 0.70
(95% CI: 0.62–0.77)
Fleiss' κ (95% CI)
Developed
Transitional
I
Transitional basophilic fibers
Pairwise Cohen's κ
Pairwise percent agreement
Developed basophilic fibers
Rater-1
Rater-2
Rater-3
Rater-4
Cohen's κ
% agree
J
Basophilic fiber (Transitional)
Basophilic fiber (Developed)
Enlarged fiber
Count
CTRL-EDL
KO-EDL
CTRL-SOL
KO-SOL

FigureS1

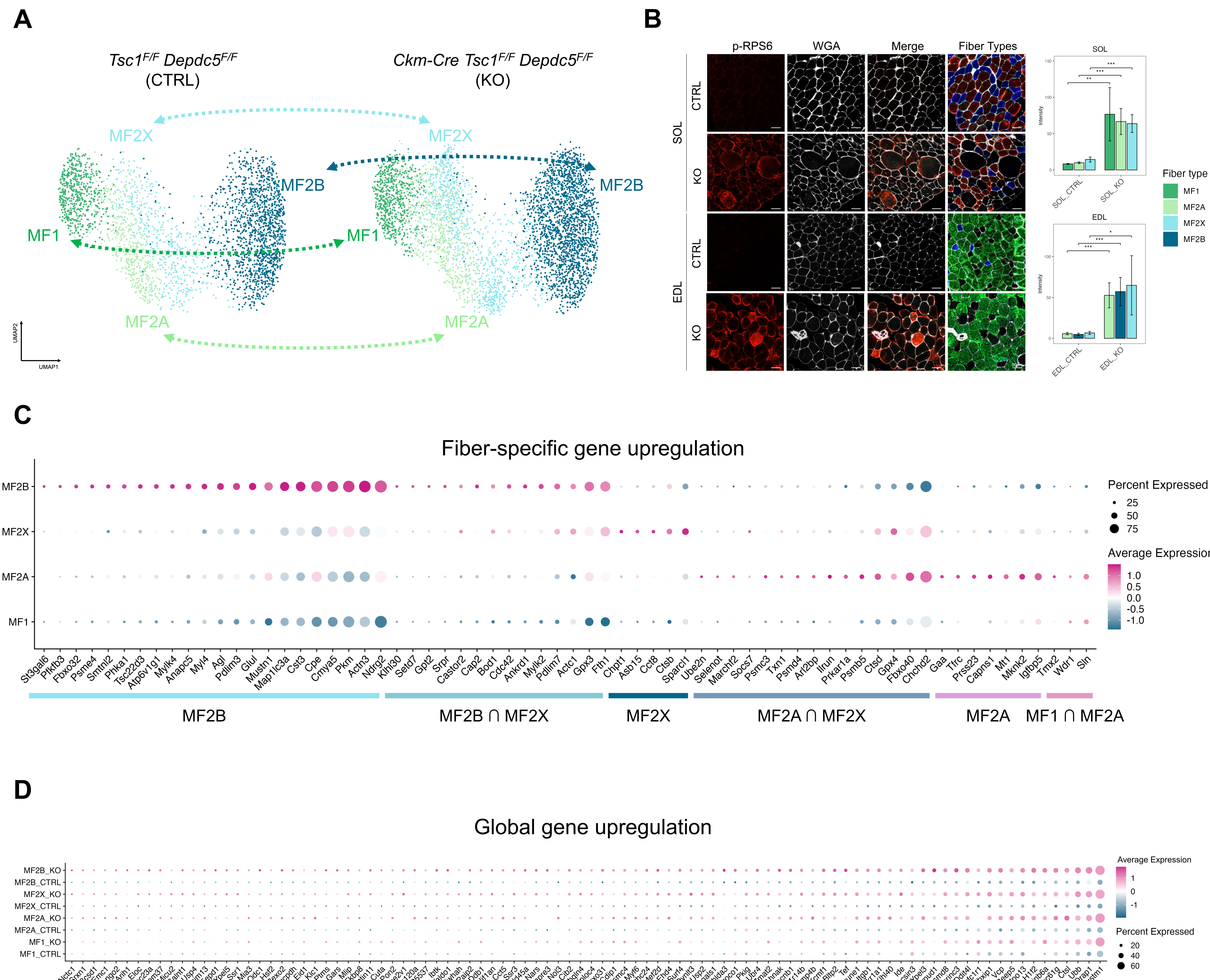
A
B
Tsc1F/F Depdc5F/F
(CTRL)
Ckm-Cre Tsc1F/F Depdc5F/F
(KO)
MF2X
MF2B
MF1
MF2A
UMAP2
UMAP1
p-RPS6
WGA
Merge
Fiber Types
SOL
EDL
CTRL
KO
Fiber type
MF1
MF2A
MF2X
MF2B
C
Fiber-specific gene upregulation
Percent Expressed
Average Expression
MF2B ∩ MF2X
MF2A ∩ MF2X
MF1 ∩ MF2A
D
Global gene upregulation

FigureS2

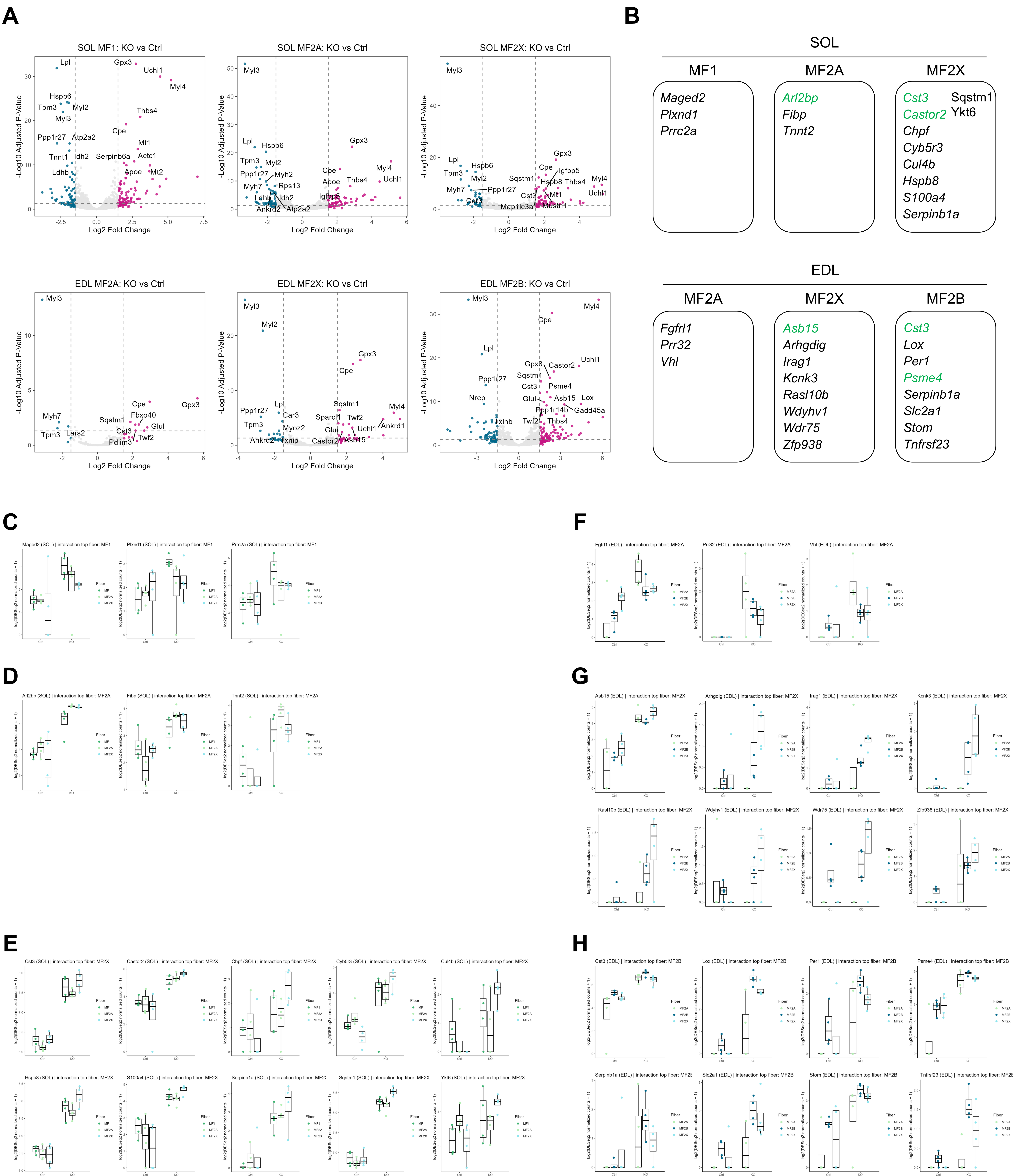
A
SOL MF1: KO vs Ctrl
SOL MF2A: KO vs Ctrl
SOL MF2X: KO vs Ctrl
EDL MF2A: KO vs Ctrl
EDL MF2X: KO vs Ctrl
EDL MF2B: KO vs Ctrl
Log2 Fold Change
-Log10 Adjusted P-Value
B
SOL
MF1
Maged2
Plxnd1
Prrc2a
MF2A
Arl2bp
Fibp
Tnnt2
MF2X
Cst3
Castor2
Chpf
Cyb5r3
Cul4b
Hspb8
S100a4
Serpinb1a
Sqstm1
Ykt6
EDL
MF2A
Fgfrl1
Prr32
Vhl
MF2X
Asb15
Arhgdig
Irag1
Kcnk3
Rasl10b
Wdyhv1
Wdr75
Zfp938
MF2B
Cst3
Lox
Per1
Psme4
Serpinb1a
Slc2a1
Stom
Tnfrsf23
C
D
E
F
G
H

FigureS3

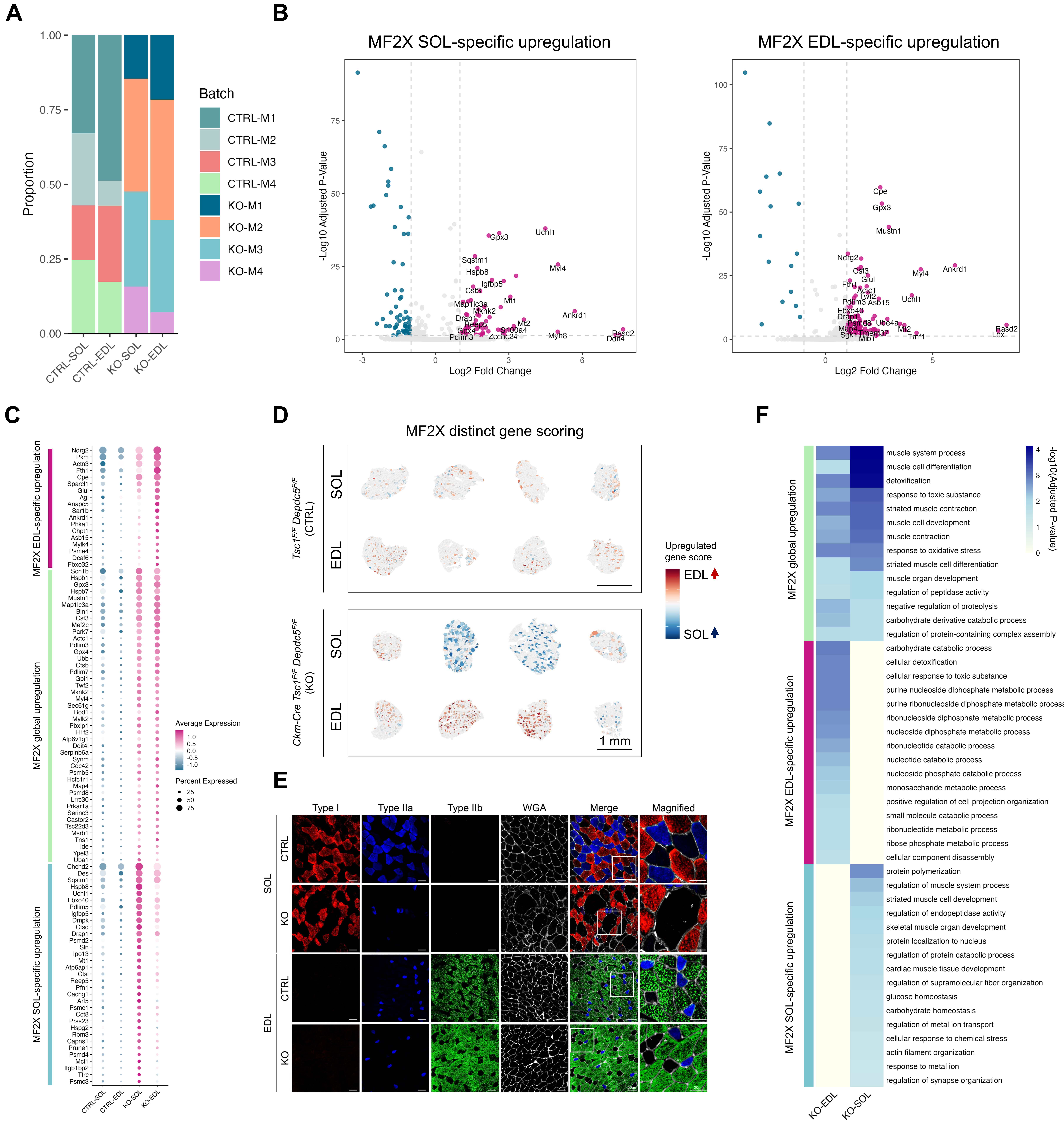
A
B
MF2X SOL-specific upregulation
MF2X EDL-specific upregulation
Proportion
Batch
CTRL-M1
CTRL-M2
CTRL-M3
CTRL-M4
KO-M1
KO-M2
KO-M3
KO-M4
CTRL-SOL
CTRL-EDL
KO-SOL
KO-EDL
-Log10 Adjusted P-Value
Log2 Fold Change
C
D
MF2X distinct gene scoring
SOL
EDL
Upregulated gene score
1 mm
E
Type I
Type IIa
Type IIb
WGA
Merge
Magnified
CTRL
KO
F
MF2X global upregulation
MF2X EDL-specific upregulation
MF2X SOL-specific upregulation
Average Expression
Percent Expressed
-log10(Adjusted P-value)
KO-EDL
KO-SOL

FigureS4

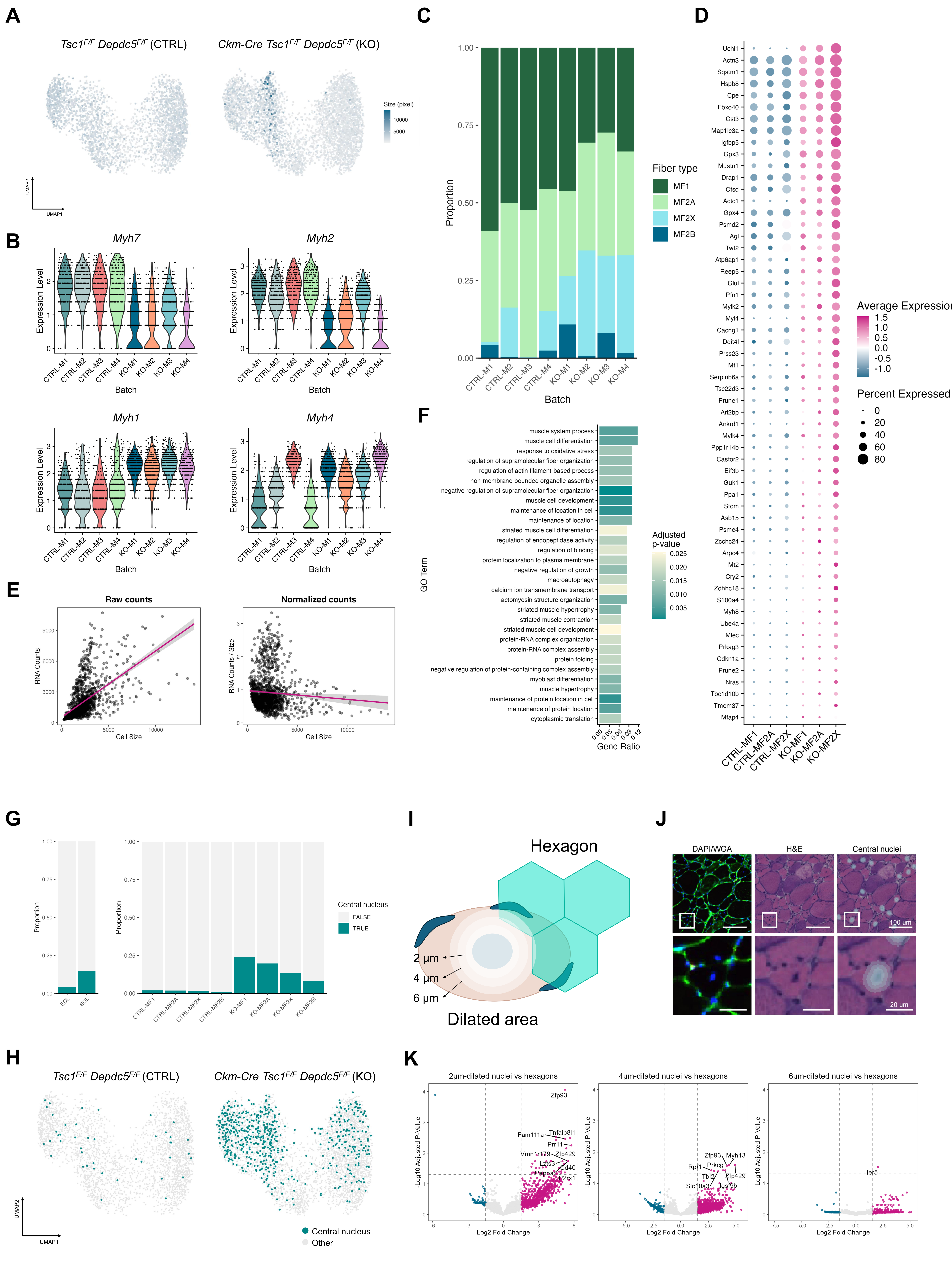

A
Tsc1F/F Depdc5F/F (CTRL)
Ckm-Cre Tsc1F/F Depdc5F/F (KO)
Size (pixel)
10000
5000
UMAP1
UMAP2
B
Myh7
Myh2
Myh1
Myh4
Expression Level
Batch
C
Proportion
Fiber type
MF1
MF2A
MF2X
MF2B
Batch
D
Average Expression
Percent Expressed
E
Raw counts
Normalized counts
RNA Counts
RNA Counts / Size
Cell Size
F
GO Term
Adjusted p-value
Gene Ratio
muscle system process
muscle cell differentiation
response to oxidative stress
regulation of supramolecular fiber organization
regulation of actin filament-based process
non-membrane-bounded organelle assembly
negative regulation of supramolecular fiber organization
muscle cell development
maintenance of location in cell
maintenance of location
striated muscle cell differentiation
regulation of endopeptidase activity
regulation of binding
protein localization to plasma membrane
negative regulation of growth
macroautophagy
calcium ion transmembrane transport
actomyosin structure organization
striated muscle hypertrophy
striated muscle contraction
striated muscle cell development
protein-RNA complex organization
protein-RNA complex assembly
protein folding
negative regulation of protein-containing complex assembly
myoblast differentiation
muscle hypertrophy
maintenance of protein location in cell
maintenance of protein location
cytoplasmic translation
G
Proportion
Central nucleus
FALSE
TRUE
H
Tsc1F/F Depdc5F/F (CTRL)
Ckm-Cre Tsc1F/F Depdc5F/F (KO)
Central nucleus
Other
I
Hexagon
2 µm
4 µm
6 µm
Dilated area
J
DAPI/WGA
H&E
Central nuclei
100 um
20 um
K
2µm-dilated nuclei vs hexagons
4µm-dilated nuclei vs hexagons
6µm-dilated nuclei vs hexagons
-Log10 Adjusted P-Value
Log2 Fold Change

FigureS5

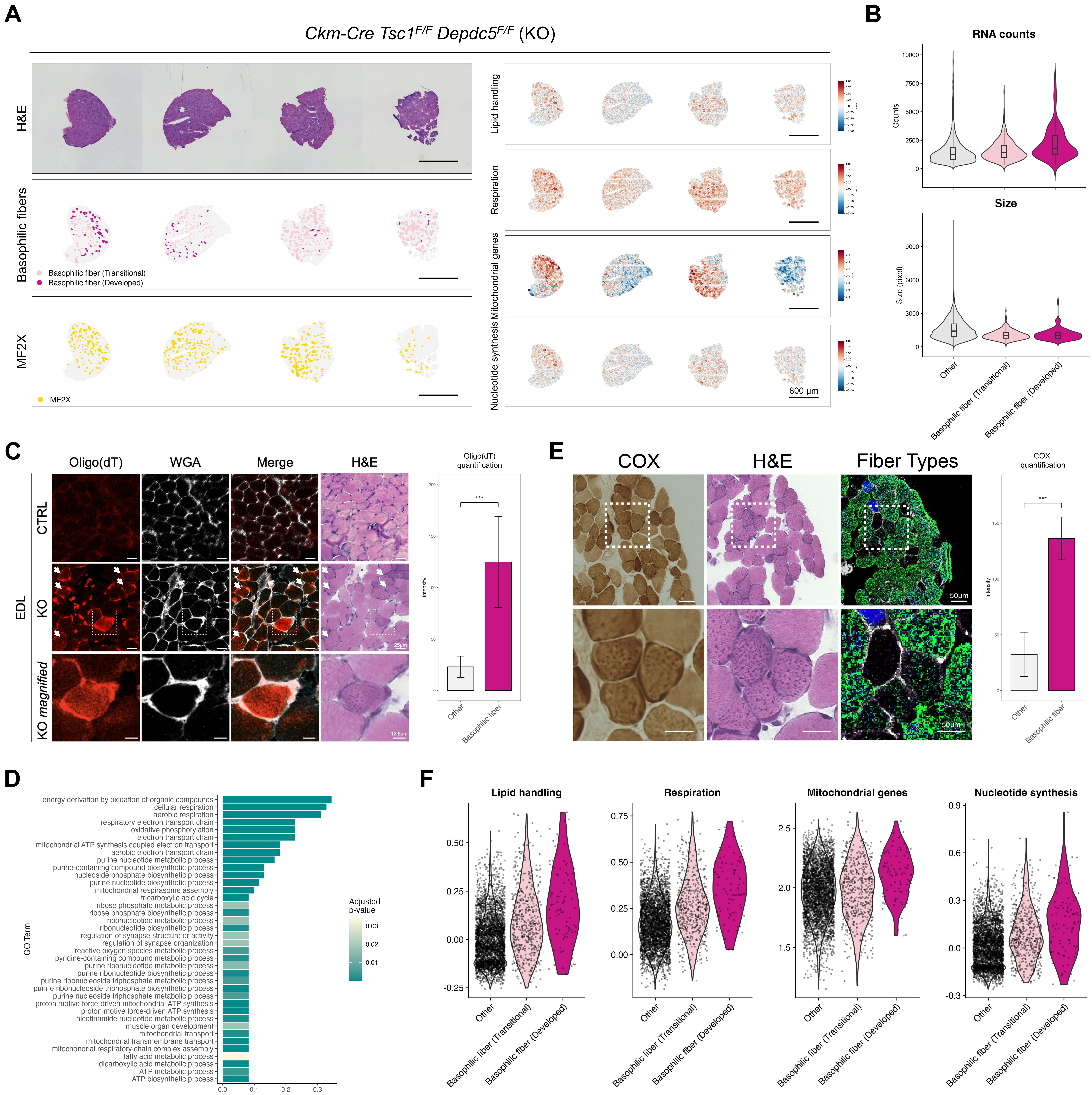
A
Ckm-Cre Tsc1F/F Depdc5F/F (KO)
H&E
Basophilic fibers
Basophilic fiber (Transitional)
Basophilic fiber (Developed)
MF2X
MF2X
Lipid handling
Respiration
Mitochondrial genes
Nucleotide synthesis
800 µm
B
RNA counts
Counts
Size
Size (pixel)
Other
Basophilic fiber (Transitional)
Basophilic fiber (Developed)
C
Oligo(dT)
WGA
Merge
H&E
EDL
CTRL
KO
KO magnified
Oligo(dT) quantification
Intensity
Other
Basophilic fiber
E
COX
H&E
Fiber Types
50µm
COX quantification
Intensity
Other
Basophilic fiber
D
energy derivation by oxidation of organic compounds
cellular respiration
aerobic respiration
respiratory electron transport chain
oxidative phosphorylation
electron transport chain
mitochondrial ATP synthesis coupled electron transport
aerobic electron transport chain
purine nucleotide metabolic process
purine-containing compound biosynthetic process
nucleoside phosphate biosynthetic process
purine nucleotide biosynthetic process
mitochondrial respirasome assembly
tricarboxylic acid cycle
ribose phosphate metabolic process
ribose phosphate biosynthetic process
ribonucleotide metabolic process
ribonucleotide biosynthetic process
regulation of synapse structure or activity
regulation of synapse organization
reactive oxygen species metabolic process
pyridine-containing compound metabolic process
purine ribonucleotide metabolic process
purine ribonucleotide biosynthetic process
purine ribonucleoside triphosphate metabolic process
purine ribonucleoside triphosphate biosynthetic process
purine nucleoside triphosphate metabolic process
proton motive force-driven mitochondrial ATP synthesis
proton motive force-driven ATP synthesis
nicotinamide nucleotide metabolic process
muscle organ development
mitochondrial transport
mitochondrial transmembrane transport
mitochondrial respiratory chain complex assembly
fatty acid metabolic process
dicarboxylic acid metabolic process
ATP metabolic process
ATP biosynthetic process
GO Term
Gene Ratio
Adjusted p-value
F
Lipid handling
Respiration
Mitochondrial genes
Nucleotide synthesis
Other
Basophilic fiber (Transitional)
Basophilic fiber (Developed)

FigureS6

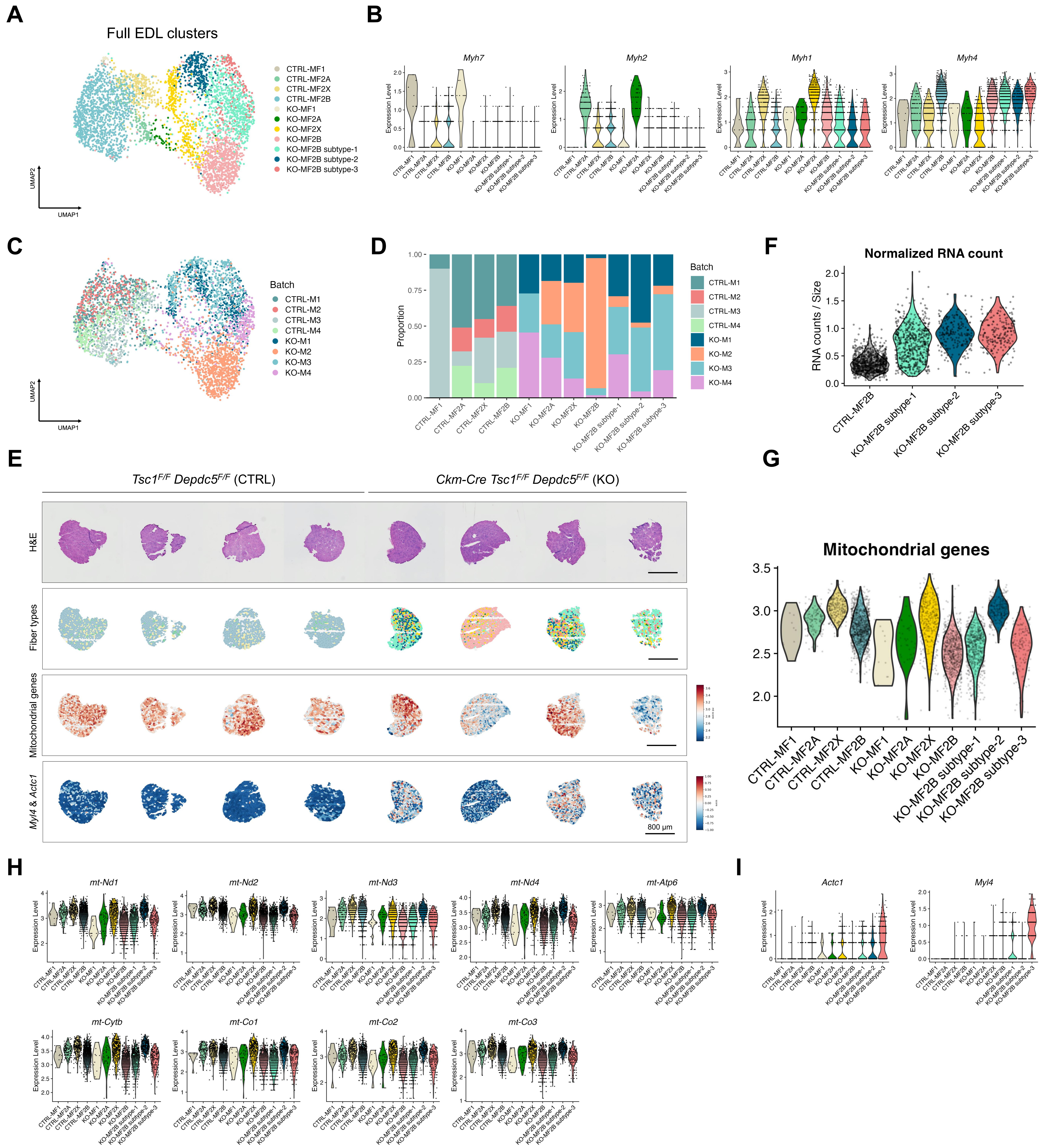
A
Full EDL clusters
CTRL-MF1
CTRL-MF2A
CTRL-MF2X
CTRL-MF2B
KO-MF1
KO-MF2A
KO-MF2X
KO-MF2B
KO-MF2B subtype-1
KO-MF2B subtype-2
KO-MF2B subtype-3
UMAP1
UMAP2
B
Myh7
Myh2
Myh1
Myh4
Expression Level
C
Batch
CTRL-M1
CTRL-M2
CTRL-M3
CTRL-M4
KO-M1
KO-M2
KO-M3
KO-M4
D
Proportion
F
Normalized RNA count
RNA counts / Size
E
Tsc1F/F Depdc5F/F (CTRL)
Ckm-Cre Tsc1F/F Depdc5F/F (KO)
H&E
Fiber types
Mitochondrial genes
Myl4 & Actc1
800 μm
G
Mitochondrial genes
H
mt-Nd1
mt-Nd2
mt-Nd3
mt-Nd4
mt-Atp6
mt-Cytb
mt-Co1
mt-Co2
mt-Co3
I
Actc1
Myl4

FigureS7

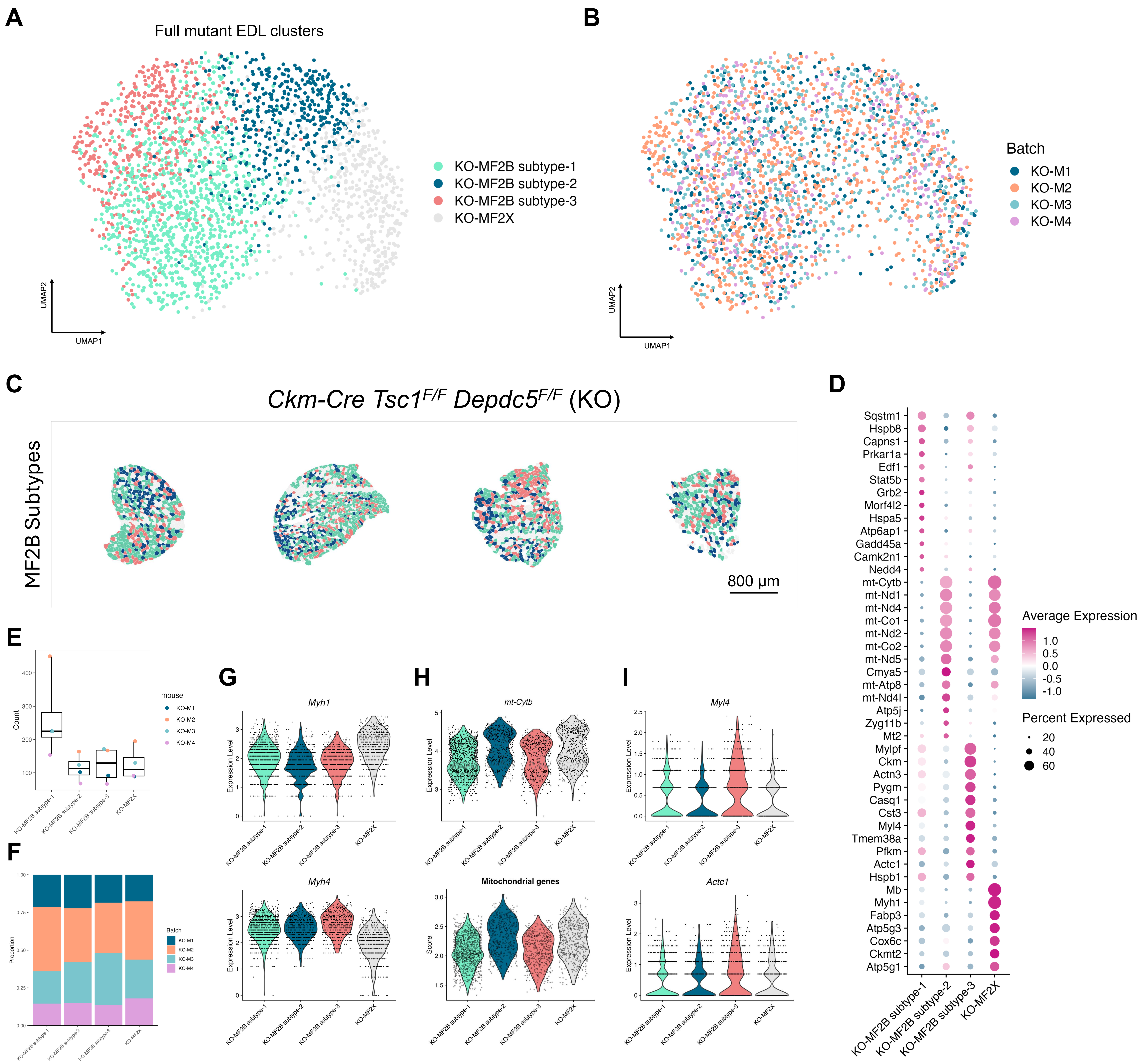
A
Full mutant EDL clusters
KO-MF2B subtype-1
KO-MF2B subtype-2
KO-MF2B subtype-3
KO-MF2X
UMAP1
UMAP2
B
Batch
KO-M1
KO-M2
KO-M3
KO-M4
C
Ckm-Cre Tsc1F/F Depdc5F/F (KO)
MF2B Subtypes
800 μm
D
Sqstm1
Hspb8
Capns1
Prkar1a
Edf1
Stat5b
Grb2
Morf4l2
Hspa5
Atp6ap1
Gadd45a
Camk2n1
Nedd4
mt-Cytb
mt-Nd1
mt-Nd4
mt-Co1
mt-Nd2
mt-Co2
mt-Nd5
Cmya5
mt-Atp8
mt-Nd4l
Atp5j
Zyg11b
Mt2
Mylpf
Ckm
Actn3
Pygm
Casq1
Cst3
Myl4
Tmem38a
Pfkm
Actc1
Hspb1
Mb
Myh1
Fabp3
Atp5g3
Cox6c
Ckmt2
Atp5g1
Average Expression
Percent Expressed
E
Count
mouse
F
Proportion
Batch
G
Myh1
Myh4
Expression Level
H
mt-Cytb
Mitochondrial genes
Score
I
Myl4
Actc1

FigureS8

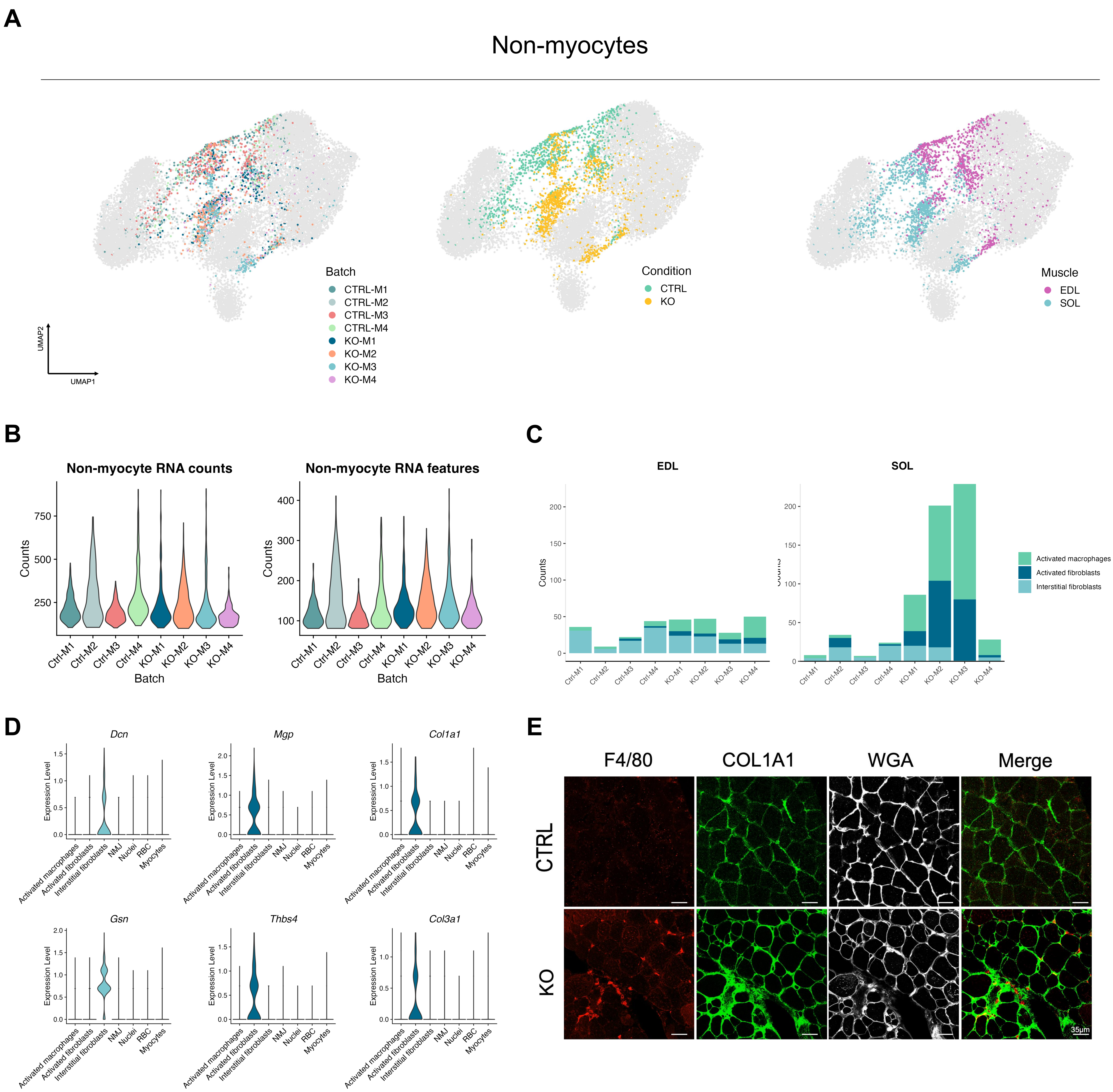
A
Non-myocytes
Batch
CTRL-M1
CTRL-M2
CTRL-M3
CTRL-M4
KO-M1
KO-M2
KO-M3
KO-M4
UMAP2
UMAP1
Condition
CTRL
KO
Muscle
EDL
SOL
B
Non-myocyte RNA counts
Non-myocyte RNA features
Counts
Batch
C
EDL
SOL
Activated macrophages
Activated fibroblasts
Interstitial fibroblasts
D
Dcn
Mgp
Col1a1
Gsn
Thbs4
Col3a1
Expression Level
E
F4/80
COL1A1
WGA
Merge
CTRL
KO
35µm

FigureS9
